# ACVR2B antagonism as a countermeasure to multi‐organ perturbations in metastatic colorectal cancer cachexia

**DOI:** 10.1002/jcsm.12642

**Published:** 2020-11-16

**Authors:** Joshua R. Huot, Fabrizio Pin, Ashok Narasimhan, Leah J. Novinger, Austin S. Keith, Teresa A. Zimmers, Monte S. Willis, Andrea Bonetto

**Affiliations:** ^1^ Department of Surgery Indiana University School of Medicine Indianapolis IN USA; ^2^ Department of Anatomy, Cell Biology and Physiology Indiana University School of Medicine Indianapolis IN USA; ^3^ Department of Otolaryngology‐Head and Neck Surgery Indiana University School of Medicine Indianapolis IN USA; ^4^ Department of Pathology and Laboratory Medicine Indiana University School of Medicine Indianapolis IN USA; ^5^ Indiana Center for Musculoskeletal Health Indiana University School of Medicine Indianapolis IN USA; ^6^ Simon Comprehensive Cancer Center Indiana University School of Medicine Indianapolis IN USA; ^7^ Zionsville Community High School Zionsville IN USA

**Keywords:** Colorectal cancer, Liver metastases, Skeletal muscle, Heart, Bone, Cachexia, Activin signalling

## Abstract

**Background:**

Advanced colorectal cancer (CRC) is often accompanied by the development of liver metastases, as well as cachexia, a multi‐organ co‐morbidity primarily affecting skeletal (SKM) and cardiac muscles. Activin receptor type 2B (ACVR2B) signalling is known to cause SKM wasting, and its inhibition restores SKM mass and prolongs survival in cancer. Using a recently generated mouse model, here we tested whether ACVR2B blockade could preserve multiple organs, including skeletal and cardiac muscle, in the presence of metastatic CRC.

**Methods:**

NSG male mice (8 weeks old) were injected intrasplenically with HCT116 human CRC cells (mHCT116), while sham‐operated animals received saline (*n* = 5–10 per group). Sham and tumour‐bearing mice received weekly injections of ACVR2B/Fc, a synthetic peptide inhibitor of ACVR2B.

**Results:**

mHCT116 hosts displayed losses in fat mass ( − 79%, *P* < 0.0001), bone mass ( − 39%, *P* < 0.05), and SKM mass (quadriceps: − 22%, *P* < 0.001), in line with reduced muscle cross‐sectional area ( − 24%, *P* < 0.01) and plantarflexion force ( − 28%, *P* < 0.05). Further, despite only moderately affected heart size, cardiac function was significantly impaired (ejection fraction %: − 16%, *P* < 0.0001; fractional shortening %: − 25%, *P* < 0.0001) in the mHCT116 hosts. Conversely, ACVR2B/Fc preserved fat mass ( + 238%, *P* < 0.001), bone mass ( + 124%, *P* < 0.0001), SKM mass (quadriceps: + 31%, *P* < 0.0001), size (cross‐sectional area: + 43%, *P* < 0.0001) and plantarflexion force ( + 28%, *P* < 0.05) in tumour hosts. Cardiac function was also completely preserved in tumour hosts receiving ACVR2B/Fc (ejection fraction %: + 19%, *P* < 0.0001), despite no effect on heart size. RNA sequencing analysis of heart muscle revealed rescue of genes related to cardiac development and contraction in tumour hosts treated with ACVR2B/Fc.

**Conclusions:**

Our metastatic CRC model recapitulates the multi‐systemic derangements of cachexia by displaying loss of fat, bone, and SKM along with decreased muscle strength in mHCT116 hosts. Additionally, with evidence of severe cardiac dysfunction, our data support the development of cardiac cachexia in the occurrence of metastatic CRC. Notably, ACVR2B antagonism preserved adipose tissue, bone, and SKM, whereas muscle and cardiac functions were completely maintained upon treatment. Altogether, our observations implicate ACVR2B signalling in the development of multi‐organ perturbations in metastatic CRC and further dictate that ACVR2B represents a promising therapeutic target to preserve body composition and functionality in cancer cachexia.

## Introduction

Colorectal cancer (CRC) remains one of the most prevalent and fatal of all cancers worldwide, with an expected 53, 200 deaths this year in the USA alone.^1^ Further, with prevalence and mortality increasing in individuals under 55 years of age, CRC is expected to remain a clinical threat.^1^ The most severe complication accompanying CRC is the development of liver metastases (LM), which happens to highly correlate with cachexia, a multi‐organ wasting syndrome that occurs in roughly 55% of CRC patients.^2^ Cachexia is a progressive cancer‐associated disease, which presents with ongoing loss of skeletal muscle mass and strength, impeding daily function of patients, reducing treatment tolerance, and ultimately elevating mortality.^3^, ^4^, ^5^, ^6^ Further, given the severe metabolic dysregulation and hypercatabolism that persists with cachexia, it is no surprise that clinical and experimental investigations have identified perturbations in several tissues besides skeletal muscle, including fat, bone, and heart.^3^, ^7^, ^8^, ^9^, ^10^ Unfortunately, viable treatment options to counteract cachexia, especially in the most advanced stages of cancer, remain elusive.

Attempting to resolve muscle wasting induced by CRC, our group and others have generated new animal models to closely resemble the clinical advanced CRC population phenotype, in particular focusing on development of LM and cachexia.^10^, ^11^, ^12^, ^13^ To the extent of identifying novel targets for therapeutic intervention to better combat cachexia induced by CRC, recent studies from our lab have demonstrated that the formation of LM exacerbates muscle wasting, heightens markers of protein catabolism, and promotes differential signalling within skeletal muscle compared with classically used subcutaneous allograft and xenograft models.^10^, ^11^ Similarly, we have also identified heightened loss of bone, fat, and heart size upon formation of LM compared with subcutaneous tumour models of CRC, demonstrating an overall exacerbated cachectic phenotype in advanced metastatic CRC.^10^, ^11^

Among the myriad factors known to contribute to skeletal muscle wasting resulting from cancer progression, members of the transforming growth factor (TGF)‐β superfamily, signalling via their binding to the activin receptor type 2B (ACVR2B), have received much attention. Moreover, as TGF‐β members such as activin A, activin B, growth differentiation factor‐11, and myostatin have been associated with or shown to induce skeletal muscle wasting in cancer and non‐cancer settings, targeting of the ACVR2B receptor has become a promising therapeutic approach in combating cachexia.^14^, ^15^, ^16^, ^17^, ^18^, ^19^, ^20^, ^21^ In line with this notion, using ACVR2B/Fc, a soluble ACVR2B synthetic peptide inhibitor, our group and others have demonstrated preservation of skeletal muscle in experimental models of lung and CRC cachexia, as well as following chronic administration of several anticancer drugs.^17^, ^22^, ^23^, ^24^ Moreover, targeting ACVR2B signalling has shown to preserve fat and bone mass and mildly preserve cardiac size in experimental models of cancer‐induced and chemotherapy‐induced muscle wasting.^17^, ^22^, ^24^

In the present study, we sought to determine whether targeting ACVR2B signalling by administration of ACVR2B/Fc was able to combat cachexia in a newly generated mouse model of CRC‐induced LM. Here, we demonstrated that administration of ACVR2B/Fc was able to preserve body weight, fat, cancellous bone, skeletal muscle mass, and skeletal muscle strength in hosts bearing metastatic human HCT116 CRC. Further, ACVR2B/Fc treatment preserved cardiac function in tumour hosts despite no effects in whole‐heart size, thereby demonstrating that targeting ACVR2B signalling may be a viable option in combating multi‐organ dysfunction in advanced metastatic CRC.

## Methods

### Cell cultures

Prior to surgical procedures, human HCT116 cells (ATCC; Manassas, VA, USA; #CRL‐247) were cultured in McCoy's medium supplemented with 10% foetal bovine serum, 1% glutamine, 1% sodium pyruvate, and 1% penicillin/streptomycin in 5% CO_2_ at 37°C and then prepared for injection. ACVR2B/Fc protein expression was induced from stable Chinese hamster ovary (CHO) cells (a kind gift from Dr Se‐Jin Lee; Jackson Laboratory, Farmington, CT) via 100 nM Cadmium exposure in serum‐free CHO media. ACVR2B/Fc was purified from the conditioned medium using protein A Sepharose, as performed previously.^25^

### Animals

All animal studies were approved by the Institutional Animal Care and Use Committee at Indiana University School of Medicine and were in compliance with the National Institutes of Health Guidelines for Use and Care of Laboratory Animals and with the 1964 Declaration of Helsinki and its later amendments. Eight‐week‐old male NOD scid gamma (NSG) (NOD‐scid/IL2Rg^null^) immunodeficient mice (In Vivo Therapeutics Core Facility, IU Simon Cancer Center, Indianapolis, IN, USA) were group‐housed (up to five per cage) within a pathogen‐free facility at IUSM's laboratory animal resource centre. Animals were randomized into four experimental groups: sham controls (S; *n* = 5), undergoing sham surgery and receiving weekly intraperitoneal (i.p.) injection of sterile saline; ACVR2B/Fc‐treated sham animals (A; *n* = 5), undergoing sham surgery and receiving weekly i.p. injection of ACVR2B/Fc peptide (10 mg/kg); mHCT116 hosts (T, *n* = 8), receiving intrasplenic injection of 1.25 × 10^5^ human HCT116 tumour cells and weekly i.p. injection of sterile saline; and ACVR2B/Fc‐treated mHCT116 hosts (T + A, *n* = 10), undergoing intrasplenic injection of 1.25 × 10^5^ HCT116 tumour cells and receiving weekly i.p. injection of ACVR2B/Fc peptide (Supporting Information, *Figure* S1). Up to 8 and 10 animals were initially enrolled in the T and T + A groups, respectively, to account for possible complications or early deaths associated with the surgical injection of tumour cells. The surgical procedure to disseminate LM of HCT116 tumour cells was performed as previously described by our group.^11^ Briefly, animals were placed under anaesthesia (2–3% isoflurane) and administered pre‐operative slow release buprenorphine.^11^ A left subcostal incision was made to expose the peritoneum, followed by a small peritoneal incision, exposing the spleen; 100 μL of sterile saline with or without HCT116 cells was injected to the lateral portion of the spleen using a 26‐gauge needle over the period of 1 min. Following injection, the spleen was re‐implanted, the peritoneum sutured, and the skin closed with surgical staples. Animal weights and (dry or wet) food consumption were monitored daily, and all animals were euthanized under light isoflurane anaesthesia 25 days following surgery. At the time of euthanasia, skeletal muscles, cardiac, fat, and liver tissues were harvested, weighed, and snap frozen in liquid nitrogen and stored at −80°C for further studies. Mouse carcasses, including a portion of liver tissue was fixed for 2 days in 10% neutral buffered formalin and then transferred into 70% ethanol, while the tibialis anterior and soleus muscles were frozen in liquid nitrogen‐cooled isopentane for histology, as previously described.^11^

### Body composition assessment

Assessment of lean (muscle) and fat (adipose) mass, that is, body composition, was performed at baseline and the day of sacrifice in un‐anaesthetized, but physically restrained mice using Echo medical systems' EchoMRI‐100 (EchoMRI, Houston, USA) as performed previously.^22^

### Whole‐body grip strength assessment

Whole‐body grip strength was assessed using a commercially available automatic grip strength metre (Columbus Instruments, Columbus, OH, USA) as previously indicated.^26^ The absolute force (expressed in grams) was recorded over five measurements, with the top three measurements utilized for analysis. To avoid habituation bias, animals were only tested at the end of the experimental period.

### *In vivo* muscle contractility

Animals (*n* = 4) underwent *in vivo* plantarflexion torque assessment (Aurora Scientific Inc., Canada), as previously described.^27^ Briefly, the left hind foot was taped to the force transducer and positioned to where the foot and tibia were aligned at 90°. The knee was then clamped at the femoral condyles, avoiding compression of the fibular nerve. Two disposable monopolar electrodes (Natus Neurology, Middleton, WI, USA) were placed subcutaneously posterior/medial to the knee in order to stimulate the tibial nerve. Peak twitch torque was first established in order to determine maximal stimulus intensity. Following determination of stimulus intensity, mice were subjected to an incremental frequency stimulation protocol to assess force–frequency relationships. The protocol utilized 0.2 ms pulses at 10, 25, 40, 60, 80, 100, 125, and 150 Hz with 1 min in between stimulations.

### Echocardiographic analysis

High‐resolution transthoracic echocardiography was performed on loosely restrained conscious mice to phenotypically characterize them 3 weeks following tumour cell injection using a Vevo 2100 Biomicroscopy system (VisualSonics, Inc., Toronto, Ontario, Canada), as previously described^28^, ^29^ (*n* = 3–5). Briefly, two‐dimensional guided M‐mode echocardiography analysis of the left ventricle (LV) was performed in the parasternal long axis at the level of the papillary muscle. Distance from the edges of the epicardium and endocardium was used to measure anterior wall thickness and posterior wall thickness in diastole and systole, as well as left ventricular internal diameters [shown as left ventricular end‐diastolic dimension (LVEDD) and left ventricular end‐systolic dimension (LVESD)]. The LV volume (LV Vol) in diastole was determined as [LV VolD: (7/2.4 + LVEDD) × LVEDD3 × 1000], whereas the LV Vol in systole was determined as [LV VolS: (7/2.4 + LVESD) × LVESD3 × 1000]. Left ventricular systolic function was assessed by ejection fraction (EF) [EF%: (LV VolD − LVVolS)/LV VolD × 100] and fractional shortening (FS) [FS%: (LVEDD/LVESD)/LVEDD × 100]. M‐mode measurements represent the average of three consecutive cardiac cycles from each mouse as previously described.^28^, ^29^ Data were analysed blinded to mouse treatment.

### Microcomputed tomography analysis of femur bone morphometry

Microcomputed tomography (μCT) scanning was performed to measure morphological indices of metaphyseal regions of femurs, as described in Bouxsein *et al*.^30^ After euthanasia, the mouse carcasses were fixed for 2 days in 10% neutral buffered formalin, transferred into 70% ethanol, the right femurs dissected, and prepared for μCT scanning on a high‐throughput μCT specimen scanner. Bone samples were rotated around their long axes, and images were acquired using a Bruker Skyscan 1176 (Bruker, Kontich, Belgium) with the following parameters: pixel size = 9 μm^3^; peak tube potential = 50 kV; X‐ray intensity = 500 μA; 0.3° rotation step. Raw images were reconstructed using SkyScan reconstruction software (NRecon; Bruker) to three‐dimensional cross‐sectional image data sets using a three‐dimensional cone beam algorithm. Structural indices were calculated on reconstructed images using the Skyscan CT Analyser software (CTAn; Bruker). Trabecular bone was separated using a custom processing algorithm in CTAn, based on the different thicknesses of the structures. Trabecular bone was analysed between 0.5 and 1.5 mm under the femoral distal growth plate using a threshold of 80–255. Trabecular parameters included bone volume fraction (BV/TV), number (Tb.N), thickness (Tb.Th), separation (Tb.Sp), pattern factor (Tb.Pf), and connectivity (Conn.Dn).

### Haematoxylin and eosin staining

In order to assess the formation of LM, liver tissue was fixed, paraffin embedded, and sectioned (10 μm) in preparation for haematoxylin and eosin staining as performed previously.^10^ Haematoxylin and eosin‐stained liver sections were then observed under an Axio Observer.Z1 motorized microscope (Zeiss, Oberchoken, Germany), and ×5 images were recorded for tumour infiltration assessment. Using ImageJ 1.43 software,^31^ tumour area relative to liver area (expressed as a percentage) was assessed.

### Muscle cross‐sectional area

To assess skeletal muscle atrophy, 10 μm‐thick cryosections taken at the mid‐belly of the tibialis anterior and soleus muscles were processed for immunostaining as described previously.^11^ Briefly, sections were blocked for 1 h at room temperature and incubated overnight at 4°C with a dystrophin (tibialis anterior: Developmental Studies Hybridoma Bank, Iowa City, IA, USA; #MANDRA1(7A10)) or laminin (soleus: MilliporeSigma, Burlington, MA, USA; #05‐206) followed by a 1 h secondary antibody (AlexaFluor 555 #A‐21227 and #A‐21434; Thermo Fisher Scientific, Waltham, MA, USA) incubation at room temperature. Entire sections were analysed for cross‐sectional area (CSA) using Lionheart LX automated microscope (BioTek Instruments, Winooski, VT, USA).

### Interleukin‐6 and insulin‐like growth factor‐1 plasma levels

Mouse interleukin (IL)‐6 and insulin‐like growth factor (IGF)‐1 were measured in EDTA‐treated mouse platelet‐poor plasma via magnetic luminex assay (IL‐6: LXSAMSM‐BR27; IGF‐1: LXSAMSM‐BR55; R&D Systems, Minneapolis, MN, USA) per the manufacturer's instructions.

### Western blotting

Protein extracts were obtained by homogenizing 50 mg of quadriceps muscle or heart tissue in RIPA buffer (150 mM NaCl, 1.0% NP‐40, 0.5% sodium deoxycholate, 0.1% SDS, and 50 mM Tris, pH 8.0) supplemented with inhibitor cocktails for proteases (Roche, Indianapolis, IN, USA) and phosphatases (Thermo Scientific, Rockford, IL, USA) on ice. Any cellular debris was removed by centrifugation (15 min, ×14 000 *g* at 4°C), and protein concentration was assessed using the BCA protein assay method (Thermo Scientific). Protein extracts (30 μg) were electrophoresed in 4–15% gradient SDS Criterion TGX precast gels (Bio‐Rad, Hercules, CA, USA) and transferred to nitrocellulose membranes (30 min at 100 V; Bio‐Rad). Membranes were blocked with odyssey blocking buffer (LI‐COR Biosciences, Lincoln, NE, USA) at room temperature for 1 h and incubated overnight in primary antibodies at 4°C with gentle rocking. Following primary antibody incubation, membranes were washed three times with PBS containing 0.2% Tween‐20 (PBST), and the membrane was incubated at room temperature for 1 h with either anti‐rabbit IgG (H + L) DyLight 800 or anti‐mouse IgG (H + L) DyLight 680 secondary antibodies (Cell Signaling Technologies, Danvers, MA, USA). Blots were again washed three times using PBST and then visualized and quantified using the Odyssey Infrared Imaging System (LI‐COR Biosciences). Antibodies used were phospho‐STAT3 (Tyr705) (#9145), STAT3 (#12640), phospho‐AKT (Ser473) (#4060), AKT (#9272), phospho‐ERK1/2 (Thr202/Tyr204) (#4370), ERK1/2 (#4695), phospho‐p38 (Thr180/Tyr182) (#4511), p38 (#9212), Ubiquitin (#3933), OPA‐1 (#80471), Mitofusin‐2 (#9482), Cytochrome C (#11940), COX IV (#4844), VDAC (#4866), and DRP1 (#8570) from Cell Signaling Technologies; PGC‐1α (#AB3242) from MilliporeSigma; PGC‐1β (#ab176328) from Abcam; and α‐Tubulin (#12G10) from Developmental Studies Hybridoma Bank. In general, phosphorylated protein levels were normalized to the expression of the respective total proteins, and tubulin was used as loading control.

### Pyruvate dehydrogenase and succinate dehydrogenase enzymatic activity

The enzymatic activities of pyruvate dehydrogenase (PDH) and succinate dehydrogenase (SDH) were measured using Colorimetric Assay Kits (MAK183 and MAK197, respectively) from MilliporeSigma as performed previously.^10^ Briefly, 10 mg of quadriceps muscle was homogenized in 100 μL of ice‐cold assay buffer, followed by centrifugation (5 min; ×10 000 *g*; 4°C); 15 μL of supernatant was added to 96‐well plates. PDH and SDH reaction mixes were added to appropriate wells, resulting in a colorimetric (450 nm for PDH and 600 nm for SDH) product proportional to the enzymatic activity. The absorbance was recorded by incubating the plate (37°C for PDH and 25°C for SDH) and taking measurements (450 and 600 nm) every 5 min for 30 and 20 min, respectively.

### Succinate dehydrogenase staining

Tibialis anterior muscles were cut into 10 μm cross‐sections on a cryostat and incubated for 30 min at 37°C with 0.5 mg/mL nitroblue tetrazoliumand (MilliporeSigma), 50 mM Na‐succinate (MilliporeSigma), and 0.08 mM phenazine methosulfate (Thermo Fisher Scientific) in PBS as previously performed.^10^ Sections were washed three times in deionized water, mounted with PBS‐glycerol (MilliporeSigma), and photographed using an Axio Observer.Z1 motorized microscope (Carl Zeiss). Entire SDH‐stained sections were quantified for integrated density using ImageJ software (NIH).

### Real‐time quantitative polymerase chain reaction

Total RNA from the fat, heart, and quadriceps muscle was extracted using the miRNeasy Mini kit (Qiagen, Valencia, CA, USA), according to the manufacturer's instructions. RNA was quantified using a Synergy H1 spectrophotometer (BioTek) and reverse transcribed to cDNA using the Verso cDNA kit (Thermo Fisher Scientific). Transcript levels were measured by real‐time PCR (Light Cycler 96, Roche) utilizing the TaqMan gene expression assay system (Life Technologies, Carlsbad, CA, USA). Expression levels for Lipe (Mm00495359_m1), Plin1 (Mm00558672_m1), Pnpla2 (Mm00503040_m1), Fasn (Mm00662319_m1), Srebf1 (Mm01306292_m1), Srebf2 (Mm00550338_m1), Atrogin‐1 (Mm00499523_m1), and Murf‐1 (Mm01185221_m1) were detected. Gene expression was normalized to TBP (Mm01277042_m1) levels using the standard 2^−ΔΔCT^ methods.

### Next‐generation RNA sequencing

Total heart RNA was evaluated for its quantity and quality using Agilent Bioanalyzer 2100. For RNA quality, an RIN number of 7 or higher is desired and was a requirement for further processing; 100 ng of total RNA was used for each sample. cDNA library preparation included mRNA purification/enrichment, RNA fragmentation, cDNA synthesis, ligation of index adaptors, and amplification, following the KAPA mRNA Hyper Prep Kit Technical Data Sheet, KR1352 – v4.17 (Roche Corporate). Each resulting indexed library was quantified, and its quality accessed by Qubit and Agilent Bioanalyzer, and multiple libraries pooled in equal molarity; 5 μL of 2 nM pooled libraries per lane were denatured, neutralized, and applied to the cBot for flow cell deposition and cluster amplification, before loading to HiSeq 4000 for 75b paired‐end sequencing (Illumina, Inc.). Approximately 30 M reads per library were generated. A Phred quality score (Q score) was used to measure the quality of sequencing. More than 90% of the sequencing reads reached Q30 (99.9% base call accuracy). The sequencing data were first assessed using FastQC (Babraham Bioinformatics, Cambridge, UK) for quality control. Then all sequenced libraries were mapped to the mouse genome (mm10) using STAR RNA‐seq aligner^32^ with the following parameter: ‘‐‐outSAMmapqUnique 60’. The reads distribution across the genome was assessed using bamutils (from ngsutils).^33^ Uniquely mapped sequencing reads were assigned to mm10 refGene genes using featureCounts (from subread)^34^ with the following parameters: ‘‐s 2 –p –Q 10’. Quality control of sequencing and mapping results was summarized using MultiQC.^35^ Genes with read count per million < 0.5 in more than 4 of the samples were removed. Differentially expressed genes (fold change ≥ 1.5, *P*‐value < 0.05) were used for constructing the heatmap using Partek® Flow® software, version 9.0.20.0417 Copyright ©; 2020 (Partek Inc., St. Louis, MO, USA). Functional enrichment analysis was performed using DAVID bioinformatics.^36^ To understand if ACVR2B/Fc has a rescue effect in cardiac function in tumour hosts, differentially expressed genes between mHCT116 and sham were compared against ACVR2B/Fc + mHCT116 vs. mHCT116 to interpret the findings. Pathways and ontology terms with *P* < 0.05 were only considered for representation. The data discussed in this publication have been deposited in NCBI's Gene Expression Omnibus and are accessible through GEO series accession number GSE149604 (https://www.ncbi.nlm.nih.gov/geo/query/acc.cgi?acc=GSE149604).^37^

### Statistics

One‐way analysis of variance tests were performed to determine differences between experimental groups. Post hoc comparisons were accomplished via a Tukey's test, with statistical significance set a priori at *P* ≤ 0.05. All statistics were performed using GraphPad Prism 8.4.1, and data are presented as means ± standard deviation.

## Results

### ACVR2B/Fc preserves body weight and fat mass in mHCT116 tumour hosts

**Figure 1:**
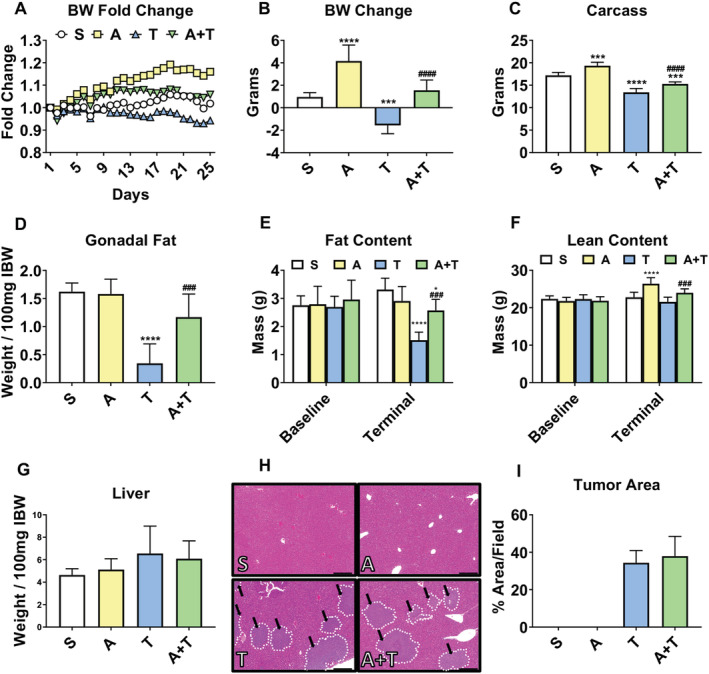
ACVR2B/Fc preserves body weight (BW) in mHCT116 hosts. (*A*) BW curves, (*B*) BW change at time of sacrifice (vs. Day 1), (*C*) carcass weights, (*D*) gonadal fat normalized to initial body weight (IBW), (*E*) fat content, and (*F*) lean content determined by EchoMRI (*E* and *F*) of NSG male mice (8 weeks old) intrasplenically injected with HCT116 tumour cells (1.25 × 10^5^ cells per mouse in sterile PBS: T) or an equal volume of vehicle (sham: S) and administered ACVR2B/Fc (*A*) (*n* = 5–10). (*G*) Liver weights normalized to IBW, (*H*) representative haematoxylin and eosin staining, and (*I*) tumour area quantification of liver tissue from S, A, T, and A + T mice. Black arrows indicate tumours, and images were taken at ×5 magnification. Scale bars: 200 μm. Data are expressed as mean ± SD. Significance of the differences: *P < 0.05, ***P < 0.001, ****P < 0.0001 vs. S; ^###^ *P* < 0.001, ^####^ *P* < 0.0001 vs. T.

Similar to our recent findings, NSG male mice bearing HCT116 LM displayed loss of body weight (*Figure* 1A and 1B), which was also accompanied by mild ascites.^11^ Carcass weights in tumour hosts (T) revealed a 22% reduction (*P* < 0.0001) compared with sham (S) animals (*Figure* 1C). ACVR2B/Fc administration to tumour hosts (A + T) was able to maintain body weight over the course of the experiment (*Figure* 1A and 1B), yielding a 14% increase (*P* < 0.0001) in carcass weight compared with T, while not having an effect on either wet or dry food consumption (*Figure* S2). Similar trends were observed for gonadal fat, which was reduced by 79% in T compared with S (*P* < 0.0001; *Figure* 1D), and for total fat at time of euthanasia, which revealed a 54% reduction in T relative to S (*P* < 0.0001; *Figure* 1E). Notably, A + T hosts displayed markedly preserved gonadal fat (+238%, *P* < 0.001) and overall terminal fat content (+70%, *P* < 0.0001) vs. T (*Figure* 1D and 1E). Interestingly, white adipose tissue gene expression levels of several markers of lipolysis (Lipe: −84%, *P* < 0.001; Plin1: −94%, *P* < 0.001; Plnpla2: −82%, *P* < 0.001) were all severely reduced in T compared with S but were all mildly preserved in A + T (Lipe: −53%, *P* < 0.05; Plin1: −68%, *P* < 0.01; Plnpla2: −56%, *P* < 0.01 vs. S) (*Figure* S3). Expression of Fasn, encoding for fatty acid synthase, was also significantly reduced in T (−89%, *P* < 0.001) compared with S and mildly preserved in A + T (−72%, *P* < 0.01 vs. S), whereas we saw no gene expression changes in either Srebf1 or Srebf2, both key players in the regulation of lipogenesis (*Figure* S3). Although the EchoMRI‐based body composition assessment did not reveal significant reductions in lean mass of T compared with S, we did observe an 11% increase (*P* < 0.01) in T + A compared with T alone (*Figure* 1F). Similar to previous findings, formation of HCT116 LM did not lead to a significant increase in liver weights in T or A + T compared with S (*Figure* 1G and 1H).^11^ Moreover, tumour area did not differ between T and A + T (*Figure* 1H and 1I).

### ACVR2B/Fc preserves skeletal muscle mass and strength in mHCT116 tumour hosts

**Figure 2:**
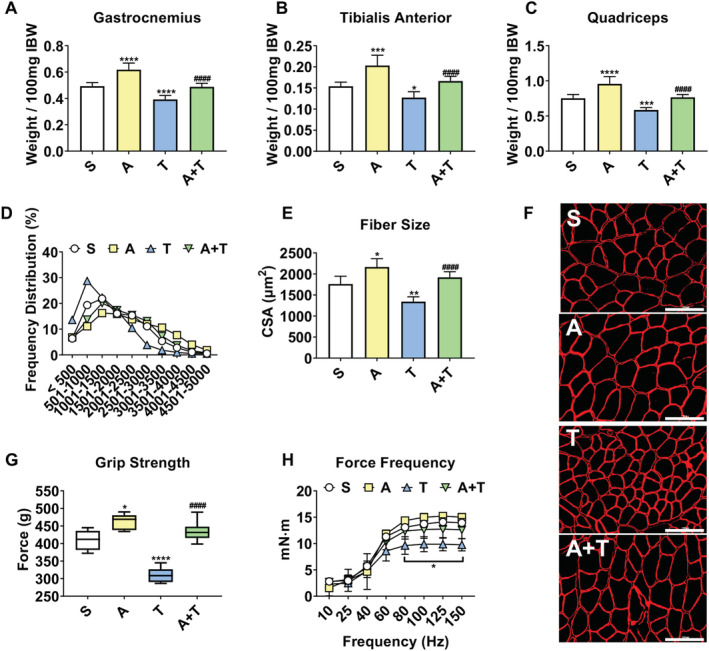
ACVR2B/Fc preserves muscle mass and strength in mHCT116 hosts. (*A*) Gastrocnemius, (*B*) tibialis anterior, and (*C*) quadriceps muscles normalized to initial body weight (IBW) in NSG male mice (8 weeks old) intrasplenically injected with HCT116 tumour cells (1.25 × 10^5^ cells per mouse in sterile PBS: T) or an equal volume of vehicle (sham: S) and administered ACVR2B/Fc (*A*) (*n* = 5–10). (*D*) Cross‐sectional area (CSA) frequency distribution, (*E*) mean CSA, and (*F*) representative images of dystrophin‐stained tibialis anterior muscles for CSA assessment (*n* = 4–6). Images were taken at ×20 magnification. Scale bars: 100 μm. (*G*) Grip strength assessment (*n* = 5–10). (*H*) *In vivo* force–frequency plantarflexion curve (*n* = 4). Data are expressed as mean ± SD. Significance of the differences: *P < 0.05, **P < 0.01, ***P < 0.001, ****P < 0.0001 vs. S; ^####^ *P* < 0.0001 vs. T. For (*H*): *P < 0.05 vs. all other groups 80–150 Hz.

Next, in line with previous literature,^17^, ^22^, ^24^, ^38^ we wanted to assess whether use of ACVR2B/Fc was able to rescue skeletal muscle atrophy and weakness also in hosts bearing CRC LM. Consistent with the trends detected in both body mass and fat mass (*Figure* 1), T mice saw significant loss of skeletal muscle mass, with gastrocnemius (−20%, *P* < 0.0001), tibialis anterior (−17%, *P* < 0.05), and quadriceps (−22%, *P* < 0.001) all exhibiting reduced size compared with S (*Figure* 2A–2C). Moreover, CSA analysis of tibialis anterior muscle revealed a shift over to smaller fibres with a 24% (*P* < 0.01) reduction in mean size in T compared with S (*Figure* 2D–2F). Similarly, CSA of the slower, oxidative soleus muscle revealed shifts over to smaller fibres with a 24% (*P* < 0.0001) loss in mean size in T compared with S (*Figure* S4). Administration of ACVR2B/Fc was able to fully preserve skeletal muscle mass, as gastrocnemius (+24%, *P* < 0.0001), tibialis anterior (+31%, *P* < 0.0001), and quadriceps (+31%, *P* < 0.0001) were all increased in A + T compared with T (*Figure* 2A–2C). In line with the rescue in skeletal muscle mass, A + T mice also saw a 43% rescue (*P* < 0.0001) in tibialis anterior CSA and a 18% rescue (*P* < 0.01) in soleus CSA compared with T alone (*Figures* 2D–2F and S4). In line with the loss of skeletal muscle mass, grip strength was reduced 24% (*P* < 0.0001) in T compared with S and preserved in A + T compared with T (+40%, *P* < 0.0001) (*Figure* 2G). Moreover, T saw significant reductions in *in vivo* plantarflexion force compared with all other groups from 80 to 150 Hz (*P* < 0.05) (*Figure* 2H).

### ACVR2B/Fc improves markers of anabolism and catabolism in skeletal muscle of mHCT116 tumour hosts

**Figure 3:**
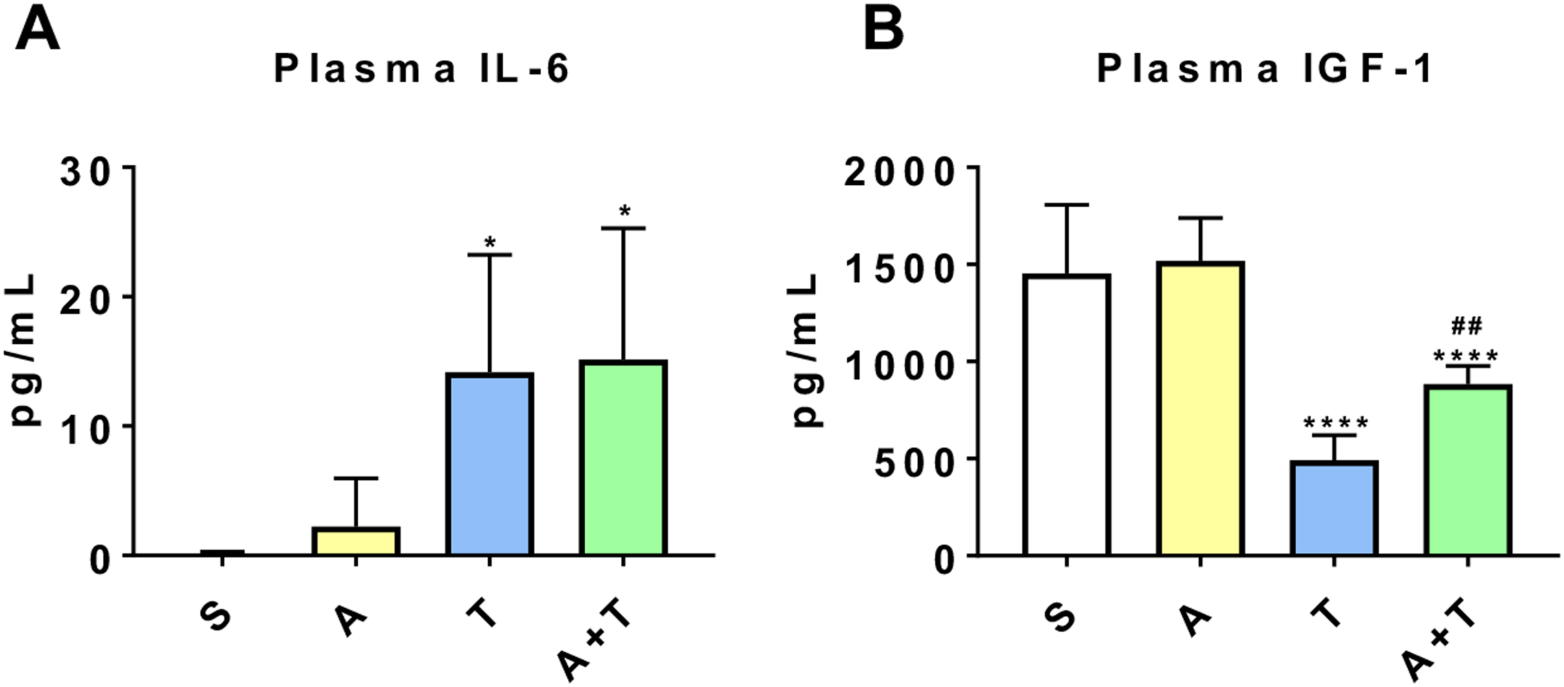
ACVR2B/Fc improves circulating IGF‐1 in mHCT116 hosts. (*A*) IL‐6 and (*B*) IGF‐1 plasma levels assessed by magnetic multiplex assay in NSG male mice (8 weeks old) intrasplenically injected with HCT116 tumour cells (1.25 × 10^5^ cells per mouse in sterile PBS: T) or an equal volume of vehicle (sham: S) and administered ACVR2B/Fc (*A*) (*n* = 5–10). Data are expressed as mean ± SD. Significance of the differences: *P < 0.05, ****P < 0.0001 vs. S; ^##^ *P* < 0.01 vs. T.

**Figure 4:**
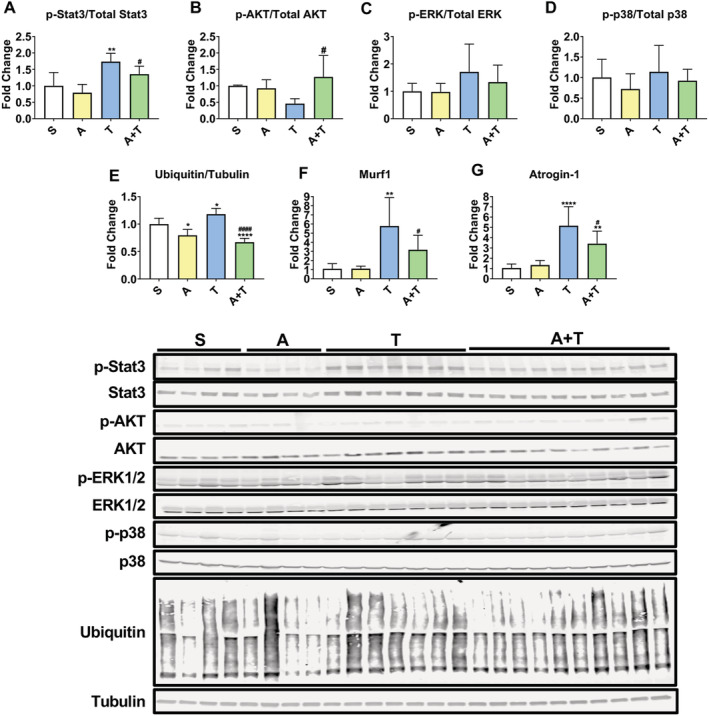
ACVR2B/Fc prevents the changes in markers of anabolism and catabolism in mHCT116 hosts. Representative western blotting and quantification (expressed as fold change vs. S) for (*A*) phospho‐Stat3, Stat3, (*B*) phospho‐AKT, AKT, (*C*) phospho‐ERK1/2, ERK1/2, (*D*) phospho‐p38, p38, (*E*) ubiquitin and tubulin from quadriceps muscle in NSG male mice (8 weeks old) intrasplenically injected with HCT116 tumour cells (1.25 × 10^5^ cells per mouse in sterile PBS: T) or an equal volume of vehicle (sham: S) and administered ACVR2B/Fc (*A*) (*n* = 4–10). Gene expression levels for (*F*) Murf1 and (*G*) Atrogin‐1 (normalized to TBP) (*n* = 5–10). Data are expressed as mean ± SD. Significance of the differences: *P < 0.05, **P < 0.01, ***P < 0.001, ****P < 0.0001 vs. S; ^#^ *P* < 0.05, ^####^ *P* < 0.0001 vs. T.

We previously demonstrated that formation of HCT116 LM increased systemic host‐derived IL‐6, and reduced host‐derived IGF‐1, in line with elevated Stat3 phosphorylation and ubiquitination within skeletal muscle.^11^ We aimed to assess whether the preservation in skeletal muscle mass upon ACVR2B/Fc administration was associated with improvement of such alterations. IL‐6 was elevated (+13.3‐fold, *P* < 0.05) in T vs. S (*Figure* 3A), consistent with increased Stat3 phosphorylation (+74%, *P* < 0.01) (*Figure* 4A). Interestingly, IL‐6 levels were not improved in A + T compared with T, although we did observe reduced levels of Stat3 phosphorylation (−22% in A + T vs. T, *P* < 0.05) (*Figures* 3A and 4A). In contrast, while IGF‐1 was reduced (−66%, *P* < 0.0001) in T animals, we did observe a rescue of circulating IGF‐1 upon administration of ACVR2B/Fc (+79%, *P* < 0.01 vs. T) (*Figure* 3B), also in line with increased AKT phosphorylation (+176%, *P* < 0.05) in the A + T mice (*Figure* 4B). Similar to our previous findings, ERK and p38 phosphorylation were unchanged in the T group, regardless of ACVR2B/Fc treatment (*Figure* 4C and 4D).^11^ In contrast, here we demonstrated that rescue of skeletal muscle mass in hosts bearing LM receiving weekly ACVR2B/Fc is associated with reductions in markers of catabolism. Indeed, while the E3 ubiquitin ligases Murf1 (+4.3‐fold *P* < 0.01 vs. S) and Atrogin‐1 (+3.9‐fold%, *P* < 0.0001 vs. S) were robustly up‐regulated in the T group, also in line with increased total ubiquitinated protein (+18%, *P* < 0.05 vs. S) (*Figure* 4E–4G),^11^ administration of ACVR2B/Fc was able to partially correct the hypercatabolic state in the muscle of mice bearing HCT116 LM, as suggested by the levels of Murf1 (−45%, *P* < 0.05 vs. T), Atrogin‐1 (−34%, *P* < 0.05 vs. T), and total ubiquitinated proteins (−43%, *P* < 0.0001 vs. T) (*Figure* 4E–4G).

### ACVR2V/Fc maintains succinate dehydrogenase enzyme activity while unaltering mitochondrial proteins in mHCT116 tumour hosts

**Figure 5:**
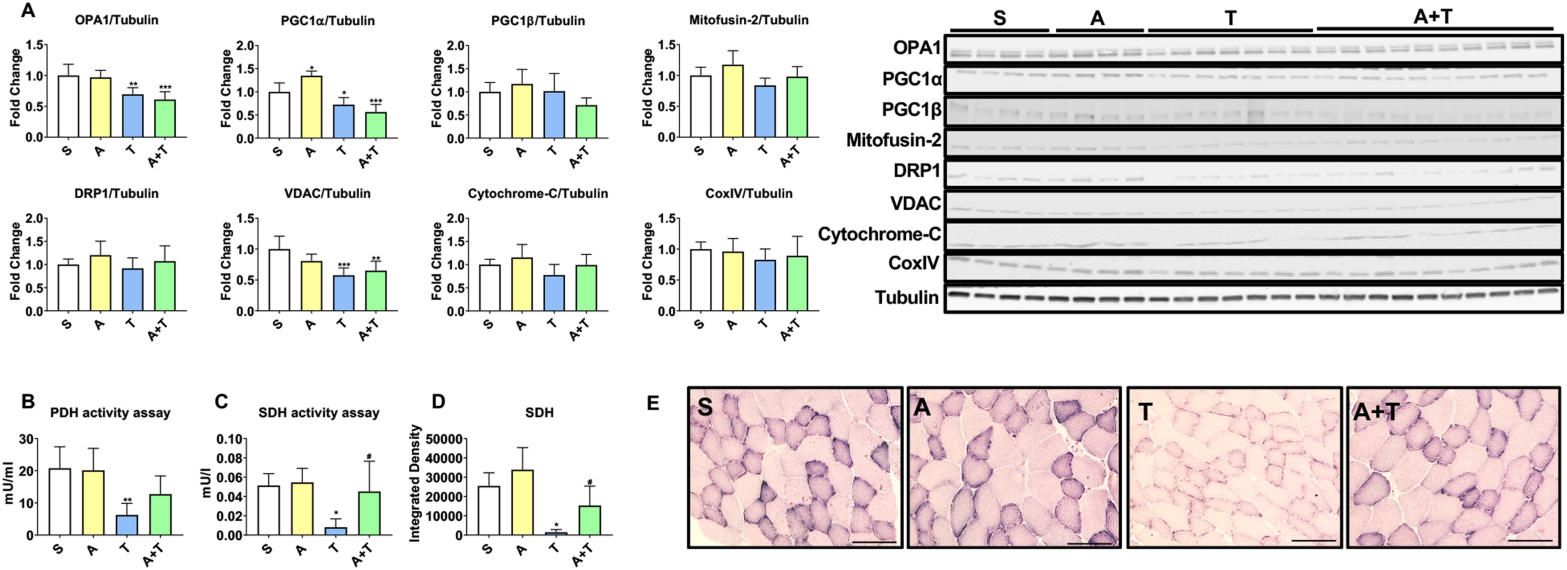
ACVR2B/Fc preserves oxidative metabolism while unaltering mitochondrial proteins in mHCT116 tumour hosts. (*A*) Representative western blotting and quantification (expressed as fold change vs. S) for OPA1, PGC1α, PGC1β, Mitofusin‐2, DRP1, VDAC, cytochrome‐C, CoxIV, and tubulin from quadriceps muscle in NSG male mice (8 weeks old) intrasplenically injected with HCT116 tumour cells (1.25 × 10^5^ cells per mouse in sterile PBS: T) or an equal volume of vehicle (sham: S) and administered ACVR2B/Fc (*A*) (*n* = 4–10). (*B*) Enzymatic activity for pyruvate dehydrogenase (PDH) and (*C*) succinate dehydrogenase (SDH) from quadriceps muscles and (*D* and *E*) SDH staining and quantification on tibialis anterior muscles (*n* = 4–6). Images were captured at a magnification of ×20. Scale bars: 100 μm. Data are expressed as means ± SD. Significance of the differences: *P < 0.05, **P < 0.01, ***P < 0.001 vs. S; ^#^ *P* < 0.05 vs. T.

Our prior work has demonstrated that skeletal muscle wasting induced by metastatic CRC is accompanied by depletion of several mitochondrial proteins as well as oxidative metabolism.^10^, ^11^ Thus, we wanted to assess whether preservation of muscle mass induced by ACVR2B antagonism was also associated with preservation of mitochondrial proteins and surrogates for oxidative metabolism. Assessment of mitochondrial proteins in the T mice demonstrated reductions in OPA1 (−31%, *P* < 0.01), PGC1α (−28%, *P* < 0.05), and VDAC (−42%, *P* < 0.001) levels, whereas PGC1β, Mitofusin‐2, DRP1, cytochrome‐C, and CoxIV were not significantly different (*Figure* 5A). Interestingly, reduced mitochondrial proteins in T mice were not restored by treatment with ACVR2B/Fc (OPA1: −39%, *P* < 0.001 vs. S; PGC1α: −44%, *P* < 0.001 vs. S; VDAC: −35%, *P* < 0.01 vs. S), suggesting that restoration of mitochondrial content is likely independent on preservation of skeletal mass resulting from blockade of ACVR2B (*Figure* 5A). In contrast, assessment of oxidative metabolism revealed improvements in A + T. Indeed, PDH enzyme activity was significantly impaired in T (−70%, *P* < 0.01) compared with S, while A + T was not significantly changed compared with S (*Figure* 5B). In line with PDH enzyme activity, SDH enzyme activity (−84%, *P* < 0.05) and SDH staining of the tibialis anterior (−94%, *P* < 0.01) were reduced in T compared with S (*Figure* 5C–5E). Conversely, SDH enzyme activity (+4.5‐fold, *P* < 0.05) and SDH staining (+9.5‐fold, *P* < 0.05) were preserved in A + T compared with T (*Figure* 5C–5E).

### ACVR2B/Fc preserves cancellous bone in mHCT116 tumour hosts

**Figure 6:**
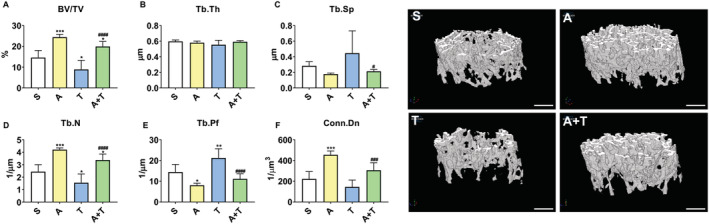
ACVR2B/Fc preserves cancellous bone in mHCT116 hosts. Representative three‐dimensional rendering of μCT scanned images and quantification of bone volume fraction (*A*) (BV/TV), (*B*) trabecular thickness (Tb.Th), (*C*) trabecular separation (Tb.Sp), (*D*) trabecular number (Tb.N), (*E*) trabecular pattern factor (Tb.Pf), and (*F*) trabecular connectivity density (Conn.Dn) of femur bones from 8‐week‐old NSG male mice (8 weeks old) intrasplenically injected with HCT116 tumour cells (1.25 × 10^5^ cells per mouse in sterile PBS: T) or an equal volume of vehicle (sham: S) and administered ACVR2B/Fc (*A*) (*n* = 4–9). Scale bars: 1 mm. Data are expressed as mean ± SD. Significance of the differences: *P < 0.05, **P < 0.01, ***P < 0.001, ****P < 0.0001 vs. S; ^#^ *P* < 0.05, ^###^ *P* < 0.001, ^####^ *P* < 0.0001 vs. T.

We have previously demonstrated that mice carrying C26 LM present with trabecular bone loss, unlike mice transplanted with C26 allografts.^9^, ^10^ To corroborate our findings in the HCT116 model of LM, a morphometric analysis of cancellous bone by μCT was performed. Similar to our previous findings, tumour‐bearing hosts displayed marked cancellous bone loss, as suggested by reduced BV/TV (−39%, *P* < 0.05 vs. T) and Tb.N (−36%, *P* < 0.05 vs. T), and by increased Tb.Pf (+47%, *P* < 0.01), whereas Tb.Th, Tb.Sp, and Conn.Dn remained substantially unchanged (*Figure* 6A–6F). Notably, consistent with our previous observations showing that ACVR2B/Fc potently counteracts chemotherapy‐induced bone loss,^22^ inhibition of ACVR2B was able to completely preserve cancellous bone measurements in tumour hosts, as suggested by augmented BV/TV (+124%, *P* < 0.0001), Tb.N (+117%, *P* < 0.0001), and Conn.Dn (+111%, *P* < 0.001), as well as by decreased Tb.Sp (−52%, *P* < 0.05) and Tb.Pf (−47%, *P* < 0.0001) (*Figure* 6A–6F).

### ACVR2B/Fc preserves cardiac function in mHCT116 tumour hosts

**Figure 7:**
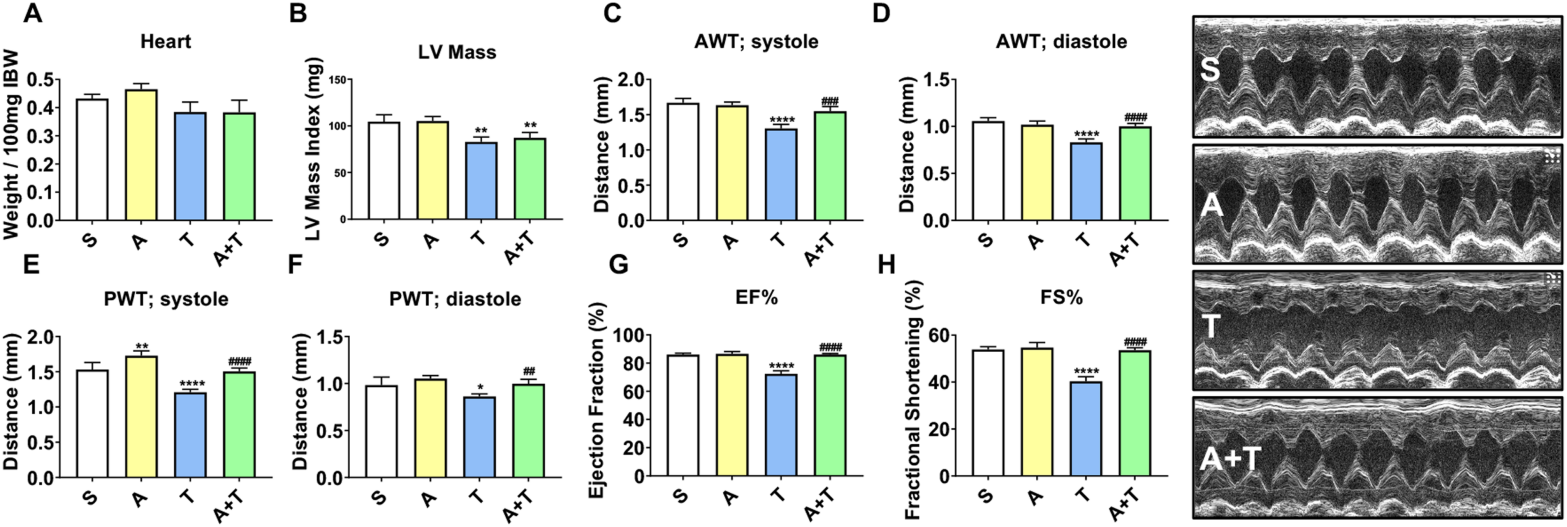
ACVR2B/Fc preserves cardiac function in mHCT116 hosts. (*A*) Heart weights normalized to initial body weight (IBW) in 8‐week‐old NSG male mice (8 weeks old) intrasplenically injected with HCT116 tumour cells (1.25 × 10^5^ cells per mouse in sterile PBS: T) or an equal volume of vehicle (sham: S) and administered ACVR2B/Fc (*A*) (*n* = 5–10). (*B*) Left ventricular (LV) mass, (*C*) anterior wall thickness (AWT) at systole, (*D*) AWT at diastole, (*E*) posterior wall thickness (PWT) at systole, (*F*) PWT at diastole, (*G*) ejection fraction percentage (EF%), and (*H*) fractional shortening percentage (FS%) determined via conscious echocardiography (*n* = 3–5). Representative images are taken from M‐mode. Data are expressed as mean ± SD. Significance of the differences: *P < 0.05, **P < 0.01, ****P < 0.0001 vs. S; ^##^ *P* < 0.01, ^###^ *P* < 0.001, ^####^ *P* < 0.0001 vs. T.

We have previously demonstrated heighted whole‐heart wasting in animals bearing CRC LM compared with traditional allograft and xenograft subcutaneous CRC implants,^10^, ^11^ although cardiac function was not taken into exam. Because CRC induces cardiovascular complications including heart failure,^39^ we documented the effects of CRC LM on cardiac function using echocardiography (Videos S5–S8) and determined if ACVR2B signalling blockade counteracted tumour‐associated cardiac dysfunction (*Figure* 7, *Table* 1). Notably, administration of ACVR2B/Fc did not exert effects on total heart mass, as we witnessed only mild whole‐heart wasting in both T (−11%, *P* = 0.09) and A + T (−11%, *P* = 0.06) compared with S (*Figure* 7A), consistent with the echocardiographic assessment of significant decreases in LV mass, with both T (−21%, *P* < 0.01) and A + T (−17%, *P* < 0.01) reduced compared with S (*Figure* 7B). Tumour hosts showed marked reductions in LV anterior wall thickness (anterior wall thickness, in systole: −22%, *P* < 0.0001, and in diastole: −21%, *P* < 0.0001; *Figure* 7C and 7D) and posterior wall thicknesses (in systole: −21%, *P* < 0.0001, and in diastole: −12%, *P* < 0.05; *Figure* 7E and 7F), consistent with cardiac muscle atrophy. Significantly decreased systolic function in the metastatic CRC group (T vs. S) was evidenced by decreased EF% (−16%, *P* < 0.0001) and FS% (−25%, *P* < 0.0001) (*Figure* 7G and 7H). Treatment of the tumour group with ACVR2B/Fc significantly protected against systolic dysfunction, evidenced by EF% and FS% (*Figure* 7G and 7H), along with measures of cardiac dilation and associated changes in volume (*Table* 1).

**Table 1:** High‐resolution transthoracic echocardiography performed on conscious mice at 3 weeks following tumour injection

Measure	Sham (S) *n* = 3	ACVR2B/Fc (A) *n* = 3	Tumour‐bearing (T) *n* = 5	A + T *n* = 5
LVEDD (mm)	2.96 ± 0.10	2.93 ± 0.099	3.06 ± 0.13	2.67 ± 0.086* ^,^ ^###^
LVESD (mm)	1.37 ± 0.078	1.33 ± 0.089	1.82 ± 0.081^****^	1.24 ± 0.040^####^
LV Vol; diastole (mL)	34.10 ± 2.89	33.17 ± 2.78	37.05 ± 3.87	26.41 ± 2.13* ^,^ ^###^
LV Vol; systole (mL)	4.82 ± 0.75	4.49 ± 0.77	10.14 ± 1.15^****^	3.68 ± 0.32^####^
LV mass/BW (mg/g)	3.77 ± 0.20	3.47 ± 0.10	3.21 ± 0.15**	3.08 ± 0.23**
HR (b.p.m.)	722 ± 9.70	683 ± 13.27*	669 ± 12.29**	686 ± 13.67**

BW, body weight; HR, heart rate; LV, left ventricle; LVEDD, left ventricular end‐diastolic dimension; LVESD, left ventricular end‐systolic dimension.

Data represent the mean ± standard deviation. A one‐way analysis of variance test was performed to determine differences between experimental groups followed by post hoc comparisons using a Tukey's test.

* *P* < 0.05.

** *P* < 0.01.

*** *P* < 0.001.

**** *P* < 0.0001 vs. S.

^##^ *P* < 0.01.

^###^ *P* < 0.001.

^####^ *P* < 0.0001 vs. T.

### ACVR2B/Fc treatment does not exhibit potent effects on cardiac signalling

Next, we sought to examine whether changes at the molecular level in the heart could mirror the effects on function, especially given that ACVR2B/Fc administration was sufficient to preserve heart function along with LV wall thickness in tumour‐bearing animals. Similar to the skeletal muscle (*Figure* 4A), we detected elevated phospho‐Stat3 ( + 69%, *P* < 0.05) in the hearts of T compared with S (*Figure* S9A), which was not corrected by ACVR2B/Fc (+71%, *P* < 0.05 vs. S) (*Figure* S9A). In contrast with data previously reported in APC^min/ +^ mice, another model for the study of CRC cachexia,^40^ the mHCT116 hosts presented reduced phosphorylation of AKT (−43%, *P* < 0.05 vs. S), which was not statistically different from S in the mice receiving the treatment (*Figure* S9B). In a similar manner, total ubiquitinated proteins were equally elevated in the T (+40%, *P* < 0.05) and A + T (+50%, *P* < 0.05) groups vs. S (*Figure* S10). Conversely, phospho‐ERK was elevated in the A (+120%, *P* < 0.05), T (+76% in T, *P* < 0.05), and A + T (+78%, *P* < 0.05) groups vs. S, whereas no alterations in the levels of either phospho‐p38 or OPA1 were detected (*Figure* S9C–S9E). Interestingly, PGC1α was found reduced in the A + T group compared with both S (−34%, *P* < 0.0001) and T (−25%, *P* < 0.01) (*Figure* S9F).

### ACVR2B/Fc treatment corrects gene networks associated with cardiac development and contraction

**Figure 8:**
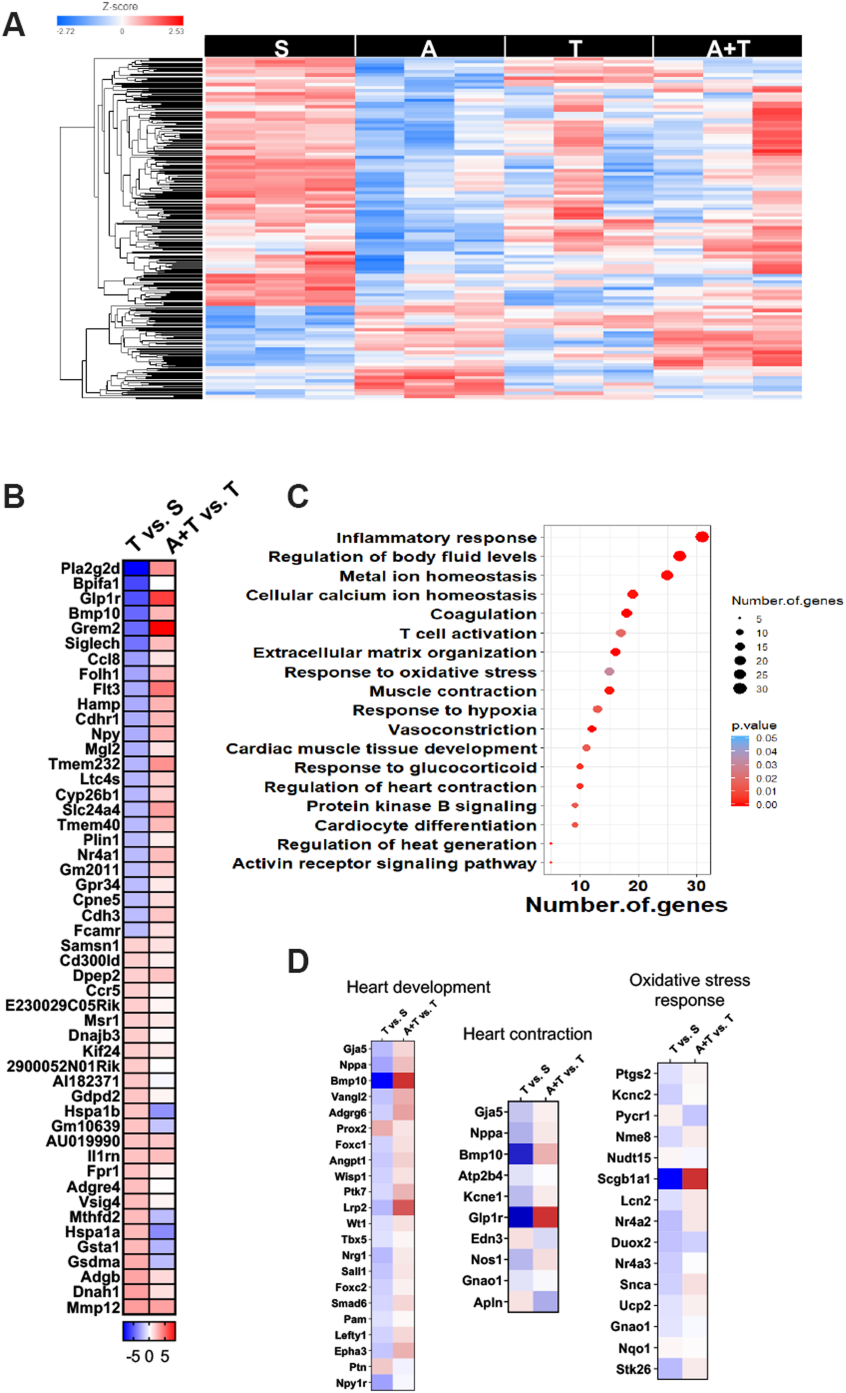
ACVR2B/Fc counteracts differentially expressed genes in hearts of mHCT116 hosts. (*A*) Heatmap of differentially expressed genes between NSG male mice (8 weeks old) intrasplenically injected with HCT116 tumour cells (1.25 × 10^5^ cells per mouse in sterile PBS: T) or an equal volume of vehicle (sham: S) and administered ACVR2B/Fc (*A*) (*n* = 3). (*B*) Top 25 up‐regulated and 25 down‐regulated differentially expressed genes in T vs. S and A + T vs. T. (*C*) Functional enrichment analysis based on the differentially expressed genes between S and T. (*D*) Differential expression of genes involved in heart development, heart contraction, and oxidative stress response in T vs. S and A + T vs. T.

We sought to take an omics approach by performing RNA sequencing to assess differences in gene signatures, which could explain the preservation of cardiac function in the HCT116 hosts administered ACVR2B/Fc. We identified 429 (fold change ≥ 1.5, *P*‐value < 0.05) differentially expressed genes in the hearts of T mice vs. S, which are reflected in the heatmap in *Figure* 8A. The complete list of differentially expressed genes are indicated in the supporting information (*Table* S11). The top 50 genes (25 up‐regulated and 25 down‐regulated) altered in T vs. S are represented in *Figure* 8B, which also demonstrates that all dysregulated genes were either reversed or the expression change was mitigated in A + T vs. T. By performing a gene ontology/functional enrichment approach on the differentially expressed genes, we identified, among others, dysregulated genes such as bone morphogenetic protein 10 (BMP10), glucagon‐like peptide 1 receptor (GLP1R), and secretoglobin family 1A member 1 (SCGB1A1), which are known to play a role in heart contraction, heart development, and oxidative stress, respectively (*Figure* 8C and 8D). These findings suggest that administration of ACVR2B/Fc was able to reverse or improve the dysregulated cardiac gene expression thereby improving cardiac function.

## Discussion

Despite recent progress in diagnosis and treatments, CRC ranks as a leader in both cancer prevalence and cancer‐related deaths in the USA.^1^ In up to 55% of CRC patients, the development of cachexia, a multi‐organ co‐morbidity of cancer, will ultimately be responsible for worsened quality of life, reduced adherence to anti‐cancer treatments, and heightened mortality.^2^, ^41^, ^42^, ^43^ In particular, loss of skeletal muscle mass, as frequently occurring during cachexia, leads to inability to perform daily functions and directly participates in over 20% of cancer‐related deaths.^41^, ^42^, ^43^ In an attempt to identify new therapeutic targets for cachexia treatment, recent studies from our group have demonstrated that formation of LM in CRC exacerbates muscle wasting by differentially affecting specific signalling networks in skeletal muscle.^10^, ^11^ Nonetheless, no approved treatment option for patients affected with cachexia is currently available.

Interestingly, a number of studies have shown that preservation of skeletal muscle mass by targeting of the ACVR2B signalling is efficacious at prolonging survival in experimental cancer cachexia.^14^, ^38^, ^44^, ^45^ Our group and others have also shown that administration of ACVR2B/Fc, a synthetic peptide and inhibitor of the ACVR2B signalling, is able to preserve skeletal muscle mass in the presence of traditional anticancer regimens, including Folfiri, cisplatin, and doxorubicin.^22^, ^24^, ^44^ Using a similar approach, here we demonstrate that ACVR2B/Fc administration in mice bearing HCT116 LM, a model for the study of advanced metastatic CRC previously generated in our laboratory,^11^ fully preserved skeletal muscle mass and strength (*Figure* 2). In line with previous observations, our findings corroborate the idea that targeting ACVR2B may be beneficial in maintaining both skeletal muscle mass and function also in advanced CRC.

It is well known that counteraction of the ACVR2B receptor disrupts the signalling mediated by several TGF‐β superfamily members and potently corrects the levels of myostatin, a negative regulator of muscle mass, within skeletal muscle in tumour‐bearing mice.^14^, ^45^ Interestingly, treatment with ACVR2B/Fc has also shown to promote muscle growth by promoting mTOR signalling and protein synthesis in cancer‐induced and chemotherapy‐induced cachexia, thereby indicating that there may be several manners in which this approach may improve skeletal muscle mass in cachectic conditions.^24^, ^44^ We and others have implicated the transcription factor Stat3 in the progression of cachexia in several experimental models, including the C26 allograft, C26 LM and Apc^min/ + ^CRC models, as well as the Lewis Lung Carcinoma, B16 melanoma, and ES‐2 ovarian cancer models.^46^, ^47^, ^48^, ^49^, ^50^, ^51^, ^52^, ^53^, ^54^, ^55^ We recently demonstrated that the IL‐6/Stat3 axis plays a role in driving HCT116‐induced muscle wasting in rodents.^11^ Here, we demonstrate that the improved muscle mass and strength upon administration of ACVR2B/Fc was also associated with improvement in Stat3 phosphorylation, further highlighting Stat3 as a pivotal prognosticator of cancer‐induced muscle wasting. Our findings also provide evidence of a Stat3–ACVR2B link in CRC‐induced cachexia (*Figure* 4), in line with results by Zhang *et al*. showing that Stat3 mediates downstream signalling of ACVR2B/activin receptor‐like kinase (ALK4) in promoting myofibroblast differentiation.^56^ Moreover, corroborating improvement in Stat3 phosphorylation, we also demonstrated ameliorated markers of protein catabolism, such as protein ubiquitination and the E3 ubiquitin ligases Atrogin‐1 and Murf‐1, which we and others have previously demonstrated in association with cachexia, including HCT116 LM (*Figure* 4).^12^, ^51^, ^57^, ^58^ Similarly, in line with the idea that unbalanced protein homeostasis (i.e. proteostasis) represents one of the hallmarks of cachexia,^3^ we and others have also shown that markers of anabolism are suppressed in mouse models for the study of CRC cachexia, as suggested by reductions of phosphorylated mTOR, 4EBP1, and p70S6K within skeletal muscle.^10^, ^59^ In line with these findings, we recently demonstrated that formation of HCT116 LM exacerbated reductions in the circulating levels of IGF‐1, a known potent anabolic signal.^11^ Interestingly, here we demonstrated that administration of ACVR2B/Fc improves circulating IGF‐1 and AKT phosphorylation in animals bearing HCT116 LM, which, at least in part, could be responsible for the preservation of skeletal muscle mass (*Figures* 3 and 4). In support of the increased circulating IGF‐1, it was recently shown that myostatin was able to reduce hepatocyte IGF‐1 production, suggesting that ACVR2B/Fc administration may preserve systemic IGF‐1 levels by antagonizing myostatin function.^60^

Over the past few years, the possibility that mitochondrial preservation may sustain skeletal muscle mass in cachectic settings has received vast attention, and preservation or overexpression of regulators involved in mitochondrial homeostasis has shown to be somewhat beneficial. Indeed, in models of CRC and lung cancer‐induced cachexia, overexpression of proteins responsible for mitochondrial fusion and biogenesis, such as Mitofusin‐2 and PGC1α, has shown to preserve skeletal muscle mass.^61^, ^62^ Moreover, modalities known to stimulate mitochondrial biogenesis such as exercise or use of exercise mimetics have also shown to maintain skeletal muscle mass in the C26 allograft model of CRC cachexia.^63^, ^64^ In the present study, we did not observe any beneficial effect associated with inhibition of ACVR2B on the expression of down‐regulated mitochondrial proteins including OPA1, PGC1α, and VDAC in tumour hosts. Perhaps more interesting, despite unchanged mitochondrial proteins, we did observe a benefit in assessment of oxidative metabolism in tumour mice treated with ACVR2B/Fc, whereby SDH enzyme activity and SDH staining of the tibialis anterior were improved, and PDH activity was unchanged in ACVR2B/Fc‐treated tumour mice compared with sham animals (*Figure* 5). This is not the first evidence that targeting ACVR2B signalling may benefit oxidative metabolism. Recent investigations have shown that citrate, citrate synthase enzyme activity, and SDH are mildly improved in mice‐exposed chemotherapy and C26 tumours.^24^, ^65^, ^66^ Interestingly, this work also demonstrated unchanged protein expression of several mitochondrial proteins indicating that targeting ACVR2B signalling may have context‐specific effects on skeletal muscle metabolism.^24^, ^65^

Recent discussion over the multi‐organ perturbations occurring with progression of cachexia has highlighted how just as skeletal muscle wasting occurs in numerous models of cancer, other organs including fat, bone, and cardiac tissue also deteriorate.^9^, ^10^, ^11^, ^51^ Interestingly, in the present study, fat wasting was ameliorated in ACVR2B/Fc‐treated tumour mice, suggesting that targeting ACVR2B is sufficient to preserve fat mass along with muscle mass, independent of food consumption. In support of this, previous findings have also demonstrated preservation of fat mass in mice treated with ACVR2B/Fc exposed to Folfiri, but the mechanisms for this remain unclear.^22^ In an attempt to provide clarity on this matter, we assessed several genes responsible for lipolysis and lipogenesis. Although we witnessed blunted reductions when compared with sham animals in genes such as Lipe, Plin1, Pnpla2, and Fasn in tumour mice treated with ACVR2B/Fc compared untreated tumour mice, there were not statistically significant differences between the two tumour groups. Of interest, Plin1 deficiency has shown to promote adipose wasting in transgenic mice, while Fasn encodes for the fatty acid synthase enzyme, a critical enzyme for *de novo* adipogenesis, thus suggesting that mild preservation in gene expression of Plin1 and Fasn in ACVRB/Fc‐treated hosts may in part explain the preservation of fat mass.^67^, ^68^ It is also important to note that systemic myostatin administration has previously shown to induce fat wasting; hence, it is possible that ACVR2B/Fc administration prevents myostatin from acting directly on adipose tissue in the tumour‐bearing mice.^69^ However, myostatin levels were not assessed in the present study. Future studies should further explore the mechanisms by which counteracting ACVR2B signalling can preserve fat mass in models of experimental cachexia.

Along the same line, similar to the exacerbated skeletal muscle wasting that occurs in models of metastatic CRC, we have also demonstrated that formation of CRC LM may exacerbate bone loss.^10^ This is of interest as recent evidence suggests that irregular muscle‐bone crosstalk may play a role in the progression of cancer cachexia. Data from our group and others have demonstrated that bone‐targeted strategies (e.g. antiresorptive treatments) preserve bone and muscle mass in the presence of platinum‐based chemotherapies.^70^, ^71^ Additionally, we have previously indicated that use of ACVR2B/Fc preserves muscle and bone in the presence of the commonly used chemotherapy regimen Folfiri.^22^ However, to our knowledge, this is the first time that ACVR2B/Fc has shown to preserve bone and muscle together in a model of metastatic CRC. Although in the present study we demonstrated complete rescue of bone mass and muscle mass with ACVR2B/Fc, chemotherapy was not included. Future studies should investigate whether targeting ACVR2B in metastatic CRC in combination with chemotherapy is sufficient for muscle–bone preservation or whether additional antiresorptive intervention is required to optimize muscle and bone health.

In line with exacerbated musculoskeletal wasting, we have also previously reported greater cardiac atrophy upon formation of LM resulting from both C26 and HCT116 CRC development,^10^, ^11^ all further corroborating the notion that cachexia is a multi‐organ wasting syndrome. However, to our knowledge, this is the first time that cardiac function has been assessed in a context of advanced experimental CRC, in line with the fact that cardiac abnormalities are known to persist in patients with CRC.^72^, ^73^, ^74^ Although in the present study we only witnessed mild whole‐heart wasting, marked reductions in LV mass and LV wall thickness, as well as impaired ventricular closing and robust declines in EF% and FS% (*Figure* 7) altogether were suggestive of cardiac cachexia. Of interest, ACVR2B/Fc administration was able to completely preserve cardiac function in the presence of metastatic CRC. In support of this observation, others have recently demonstrated that the myostatin/ACVR2B signalling is up‐regulated in the heart of patients with heart failure^75^ and that targeting ACVR2B can improve cardiac function in models of ageing, ischaemia, and myocardial infarction, thereby implicating activin signalling as a negative input on cardiac function.^76^, ^77^, ^78^ To our knowledge, ours is the first report providing evidence that ACVR2B could represent a potent therapeutic target to preserve cardiac function in the occurrence of metastatic CRC. Interestingly, cardiac function is preserved despite no effects in terms of whole‐heart size, LV mass, nor major impact on regulators of muscle growth, thereby suggesting that signalling through ACVR2B is not critical in regulating heart size in metastatic CRC. This is in line with a study by Jin *et al*., in which a growth differentiation factor‐11/myostatin inhibitor exhibited potent effects on skeletal muscle, yet no effect on heart mass.^79^ Further, similar to a study by Hulmi *et al*., which examined skeletal and cardiac muscle toxicity in response to doxorubicin, the present findings highlight potential skeletal and cardiac muscle differences when targeting ACVR2B, as both studies demonstrate a potent anabolic effect on skeletal muscle with minimal impact on cardiac size, while presently preserving cardiac function.^80^

As we only witnessed mild benefits of ACVR2B/Fc on cardiac size, yet preservation of cardiac function, our large omics approach sought to assess signalling networks that were altered by LM or influenced by treatment with ACVR2B/Fc. Interestingly, only a small number (429) of differentially expressed genes were identified in the heart of tumour hosts, yet gene ontology analysis revealed that several of the genes altered play critical roles in cardiac development and contraction (*Figure* 8). Follow‐up analysis revealed that 116 of these genes were differentially expressed in tumour hosts receiving ACVR2B/Fc compared with the untreated tumour bearers. Among these, Bmp10, which has shown to induce cardiomyocyte proliferation, preserve cardiac function following myocardial infarction, and prevent cardiomyocyte death and development of cardiac fibrosis following insult,^81^, ^82^ was down‐regulated 30‐fold in tumour hosts, whereas ACVR2B/Fc increased Bmp10 five‐fold in tumour hosts. Similarly, GLP1R was reduced 51‐fold in the tumour‐bearing mice and increased 71‐fold in ACVR2B/Fc‐treated HCT116 hosts. This is of interest, as GLP1R agonists have been implicated in preserving proper cardiac remodelling and shown to be cardioprotective following insults such as myocardial infarcts or ischaemia.^83^, ^84^, ^85^ Moreover, GLP1R has shown to be essential for HR control in mice, and thus, its severe down‐regulation in tumour hosts in this study may be responsible in part for cardiac dysfunction.^86^ Additionally, SCGB1A1, also known as uteroglobin, was reduced 70‐fold in tumour‐bearing mice and increased 44 678‐fold in tumour mice receiving ACVR2B/Fc. To date, uteroglobin has received little attention with respect to cardiac function and/or cardiovascular disease; however, loss of uteroglobin has been implicated in pulmonary inflammation and renal failure.^87^, ^88^ Further, uteroglobin is known to play an important role in combatting oxidative stress, which is known to be highly important with respect to heart failure.^89^, ^90^ Although this provides insight as to how ACVR2B/Fc may preserve cardiac function in more advanced metastatic CRC, we did not interrogate specific mechanisms of action, and thus, future studies should investigate specifically how ACVR2B/Fc administration may benefit cardiac function in models of cancer‐induced cardiac cachexia.

Overall, our study demonstrates that administration of ACVR2B/Fc is able to counteract several tissue deteriorations in a murine model of metastatic CRC. Although the present study demonstrated benefits to skeletal muscle, fat, bone, and heart, limitations include the absence of mechanistic studies to identify tissue‐specific modes of action of ACVR2B/Fc. Given the multiplicity of ligands capable of binding to ACVR2B, future studies will need to isolate which factor(s) is mainly responsible for the multi‐organ effects associated with metastatic CRC. Moreover, because of the difficulty in estimating tumour burden, based on the absence of changes in overall liver weights or liver tumour area via histological analysis, we can only speculate that administration of ACVR2B/Fc did not exert anti‐tumour effects. To validate this point, future studies will have to investigate whether pharmacologic ACVR2B blockade, alone or in combination with routinely administered anti‐cancer drugs, is sufficient to enhance the anti‐proliferative effects of chemotherapy and/or to prevent its associated musculoskeletal defects. The latter is of particular importance as we and others have demonstrated that several chemotherapies induce multi‐organ cachexia independent of their effects on tumour growth.^22^, ^23^, ^71^, ^91^, ^92^, ^93^ In this regard, targeting ACVR2B in combination with chemotherapy may prove to be a viable approach in minimizing tumour progression and the multi‐organ perturbations that occur with the progression of cancer cachexia. Moreover, the present study only examined the benefits of ACVR2B/Fc on male mice bearing HCT116 LM, and as sexual dimorphism has been observed in the progression of cachexia in CRC, future studies should investigate whether ACVR2B/Fc benefits equally span across both sexes.^49^ Lastly, the present approach demonstrates that counteraction of the ACVR2B signalling is an effective multi‐organ preservation strategy in the progression to advanced CRC; however, whether the same approach may prove effective in the rescue of tissue derangements in already established CRC cachexia remains to be determined. Of interest, a small number of clinical trials have targeted ACVR2 signalling in cachectic conditions associated with pancreatic and lung cancer (NCT01433263; NCT01505530) in an attempt to preserve lean body mass, however with minimal success. Whether these approaches are efficacious in CRC cachexia has not been reported in the literature, in particular with respect to multi‐organ preservation as demonstrated here in mice bearing CRC LM. Contrarily, novel ACVR2 ligand traps, Luspatercept and Sotatercept, are showing early promise in clinical trials in patients with myelodysplastic syndromes (NCT02631070) and pulmonary arterial hypertension (NCT03496207), yet whether either of these drugs would be beneficial in combatting the musculoskeletal complications resulting from CRC‐induced cachexia will require further testing.

In conclusion, our observations reinforced the idea that cachexia is a multi‐organ syndrome by demonstrating perturbations not just in skeletal muscle but also in fat, bone, and cardiac tissues in a model of metastatic CRC. More importantly, our data have demonstrated that targeting ACVR2B may be a promising therapeutic to counteract the multi‐organ perturbations accompanying advanced metastatic CRC, as use of ACVRB/Fc proved beneficial for the sustainment of skeletal muscle mass and strength, fat mass, bone mass, and cardiac function in mice bearing CRC LM.

## Author contributions

J.R.H. and A.B. conceived and designed the experiments; J.R.H., F.P., A.S.K., and L.J.N. performed the *in vivo* experiment and molecular characterization of cachexia; M.S.W. performed and analysed the echocardiography data; J.R.H., A.N., T.A.Z., and A.B. analysed the RNA sequencing data; J.R.H. and A.B. wrote and edited the paper.

## Conflict of interest

The authors have declared that no conflict of interest exists.

## Supporting information


**Figure S1** Schematic representation of the *in vivo* model. 8‐week old NSG male were intrasplenically injected with HCT116 tumor cells (1.25 × 10^5^ cells/mouse in sterile PBS: T) or an equal volume of vehicle (Sham: S) and administered ACVR2B/Fc (A:10 mg/kg), a synthetic peptide inhibitor of ACVR2B signaling, once weekly, intraperitoneally (i.p.). The red arrow indicates the tumor cell injection, whereas the purple arrows indicate administration of A.

**Figure S2** ACVR2B/Fc does not impact food consumption in mHCT116 hosts. (A) Wet and (B) dry food consumption in NSG male mice (8‐week old) intrasplenically injected with HCT116 tumor cells (1.25 × 10^5^ cells/mouse in sterile PBS: T) or an equal volume of vehicle (Sham: S) and administered ACVR2B/Fc (A) (*n* = 5–10).

**Figure S3** ACVR2B/Fc minimally alters gene expression in markers of lipolysis and lipogenesis in mHCT116 hosts. Gene expression for (A) Lipe, (B) Plin1, (C) Pnpla2, (D) Fasn, (E) Srebf1, and (F) Srebf2 (normalized to TBP) in NSG male mice (8‐week old) intrasplenically injected with HCT116 tumor cells (1.25 × 10^5^ cells/mouse in sterile PBS: T) or an equal volume of vehicle (Sham: S) and administered ACVR2B/Fc (A) (*n* = 4–6). Data are expressed as mean ± SD. Significance of the differences: *p < 0.05, **p < 0.01, ***p < 0.001 vs. S.

**Figure S4** ACVR2B/Fc preserves fiber size of soleus muscles in mHCT116 hosts. (A) Cross‐sectional area (CSA) frequency distribution, (B) mean CSA, and (C) representative images of laminin stained soleus muscles to assess CSA in NSG male mice (8‐week old) intrasplenically injected with HCT116 tumor cells (1.25 × 10^5^ cells/mouse in sterile PBS: T) or an equal volume of vehicle (Sham: S) and administered ACVR2B/Fc (A) (*n* = 4–6). Images were taken at 20× magnification. Scale bars: 100 μm. Data are expressed as mean ± SD. Significance of the differences: ***p < 0.001, ****p < 0.0001 vs. S; ##*p* < 0.01 vs. T.

**Video S5** Representative video of B‐mode echocardiography in S mice. Representative videos of B mode analysis determined via conscious echocardiography in NSG male sham (S) mice (8‐week old) (*n* = 3).

**Video S6** Representative video of B‐mode echocardiography in A mice. Representative videos of B mode analysis determined via conscious echocardiography in NSG male mice (8‐week old) administered ACVR2B/Fc (A) (*n* = 3).

**Video S7** Representative video of B‐mode echocardiography in T mice. Representative videos of B mode analysis determined via conscious echocardiography in NSG male mice (8‐week old) intrasplenically injected with HCT116 tumor cells (1.25 × 10^5^ cells/mouse in sterile PBS: T) (*n* = 5).

**Video S8** Representative video of B‐mode echocardiography in A + T mice. Representative videos of B mode analysis determined via conscious echocardiography in NSG male mice (8‐week old) intrasplenically injected with HCT116 tumor cells (1.25 × 10^5^ cells/mouse in sterile PBS: T) and administered ACVR2B/Fc (A) (*n* = 5).

**Figure S9** ACVR2B/Fc does not improve markers of anabolism or catabolism in the heart of mHCT116 hosts. Representative western blotting and quantification (expressed as fold change versus sham) for (A) phospho‐Stat3, Stat3, (B) phospho‐AKT, AKT, (C) phospho‐ERK1/2, ERK1/2, (D) phospho‐p38, p38, (E) OPA1, (F) PGC1α and tubulin in heart tissue from NSG male mice (8‐week old) intrasplenically injected with HCT116 tumor cells (1.25 × 10^5^ cells/mouse in sterile PBS: T) or an equal volume of vehicle (Sham: S) and administered ACVR2B/Fc (A) (*n* = 5–8). Data are expressed as mean ± SD. Significance of the differences: *p < 0.05 vs. S.

**Figure S10** Heart Ubiquitin does not improve with ACVR2B/Fc in mHCT116 hosts. Representative western blotting and quantification (expressed as fold change versus sham) for total ubiquitin and tubulin from heart tissue of 8‐week‐old NSG male mice (8‐week old) intrasplenically injected with HCT116 tumor cells (1.25 × 10^5^ cells/mouse in sterile PBS: T) or an equal volume of vehicle (Sham: S) and administered ACVR2B/Fc (A) (*n* = 5–8). Data are expressed as mean ± SD. Significance of the differences: *p < 0.05 vs. S.

**Table S11** Complete list of differentially expressed genes in hearts. List of the 429 differentially expressed genes between NSG male mice (8‐week old) intrasplenically injected with HCT116 tumor cells (1.25 × 10^5^ cells/mouse in sterile PBS: T) or an equal volume of vehicle (Sham: S) and administered ACVR2B/Fc (A) (*n* = 3).
