# Supplementary figures and images for: ACVR2B antagonism as a countermeasure to multi‐organ perturbations in metastatic colorectal cancer cachexia
Source: J Cachexia Sarcopenia Muscle. 2020 Nov 16;11(6):1779–98. doi: 10.1002/jcsm.12642 (PMC7749603; doi:10.1002/jcsm.12642)

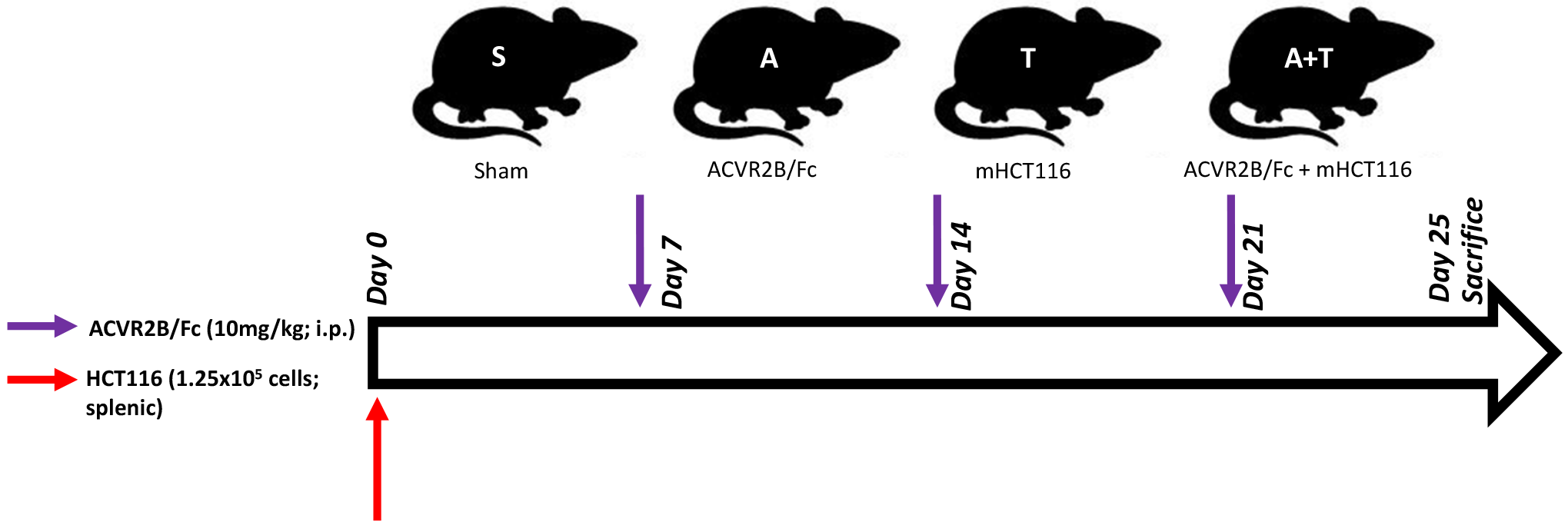

Supplement: Supplementary file 1 — Figure S1 Schematic representation of the in vivo model. 8‐week old NSG male were intrasplenically injected with HCT116 tumor cells (1.25 × 10^5^ cells/mouse in sterile PBS: T) or an equal volume of vehicle (Sham: S) and administered ACVR2B/Fc (A:10 mg/kg), a synthetic peptide inhibitor of ACVR2B signaling, once weekly, intraperitoneally (i.p.). The red arrow indicates the tumor cell injection, whereas the purple arrows indicate administration of A. [file JCSM-11-1779-s001.tiff]

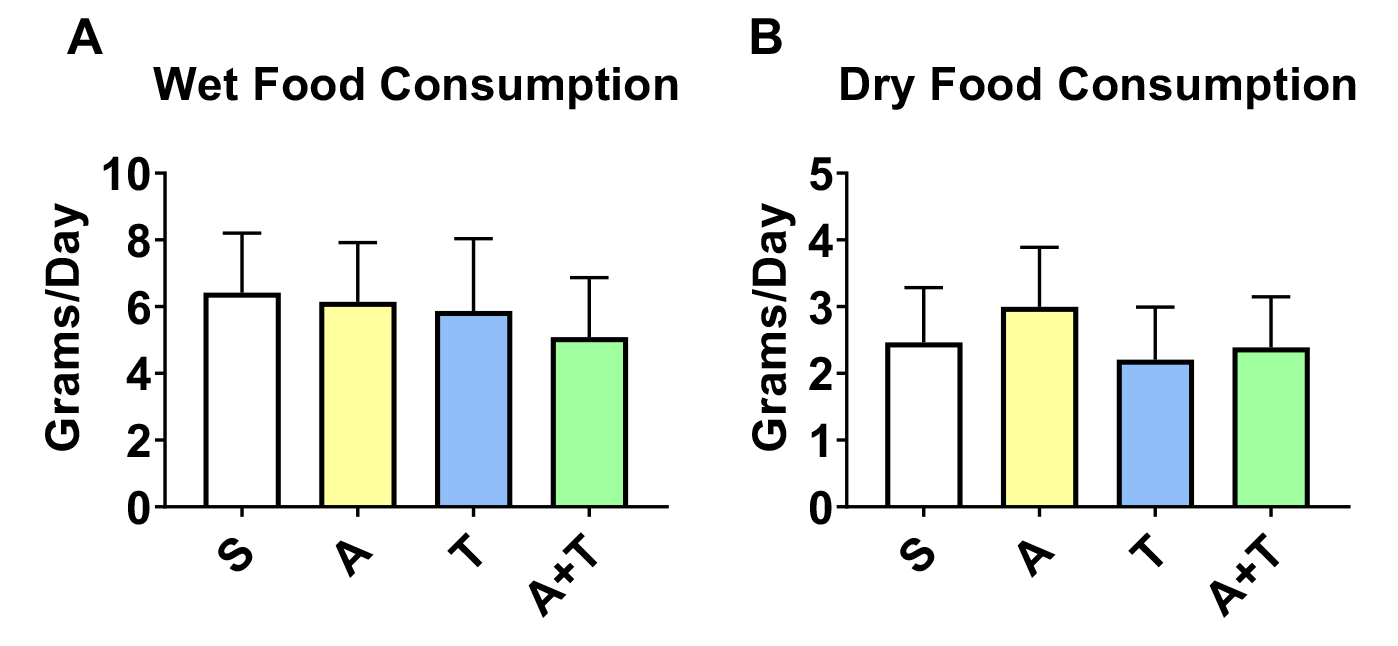

Supplement: Supplementary file 2 — Figure S2 ACVR2B/Fc does not impact food consumption in mHCT116 hosts. (A) Wet and (B) dry food consumption in NSG male mice (8‐week old) intrasplenically injected with HCT116 tumor cells (1.25 × 10^5^ cells/mouse in sterile PBS: T) or an equal volume of vehicle (Sham: S) and administered ACVR2B/Fc (A) (n = 5–10). [file JCSM-11-1779-s002.tiff]

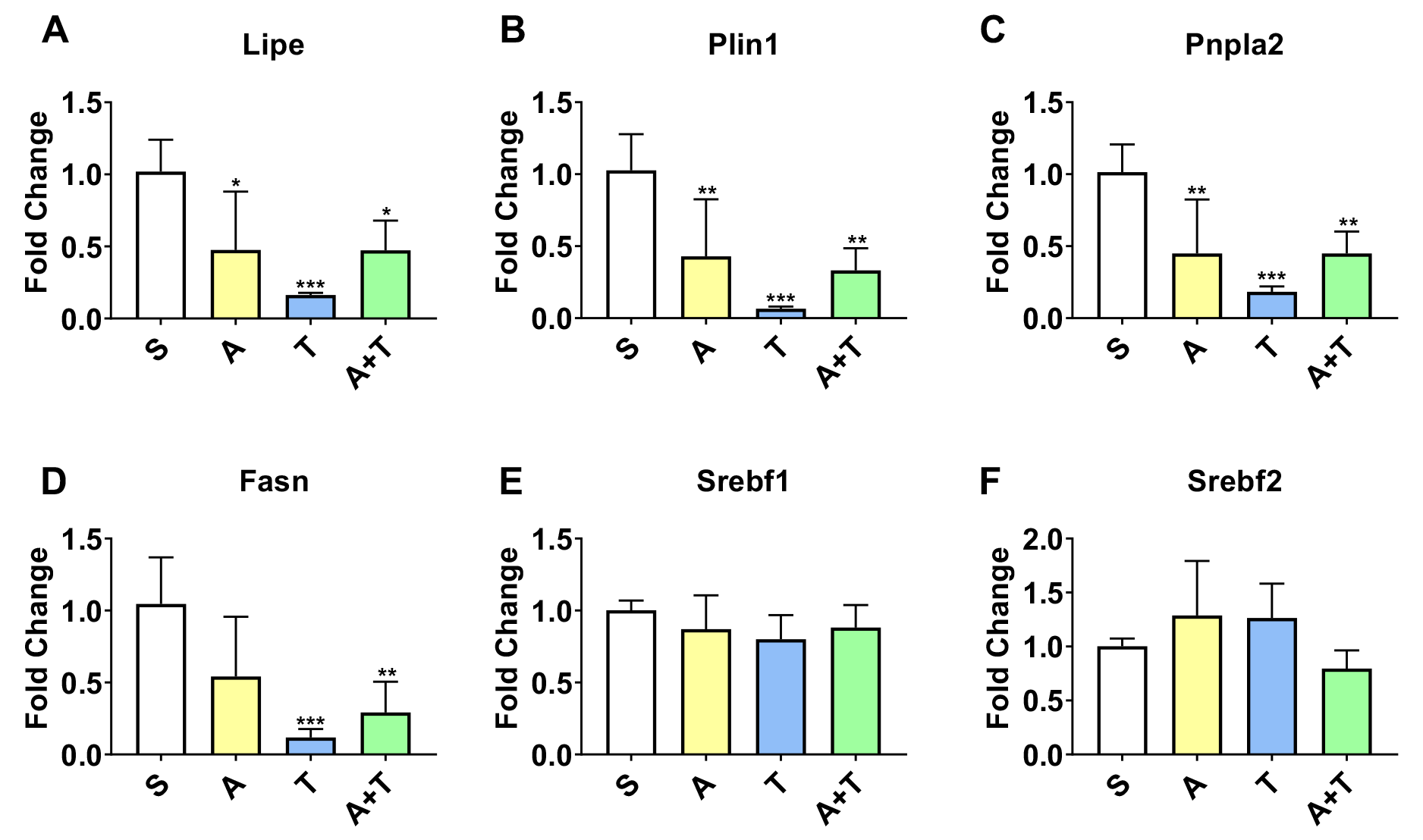

Supplement: Supplementary file 3 — Figure S3 ACVR2B/Fc minimally alters gene expression in markers of lipolysis and lipogenesis in mHCT116 hosts. Gene expression for (A) Lipe, (B) Plin1, (C) Pnpla2, (D) Fasn, (E) Srebf1, and (F) Srebf2 (normalized to TBP) in NSG male mice (8‐week old) intrasplenically injected with HCT116 tumor cells (1.25 × 10^5^ cells/mouse in sterile PBS: T) or an equal volume of vehicle (Sham: S) and administered ACVR2B/Fc (A) (n = 4–6). Data are expressed as mean ± SD. Significance of the differences: *p < 0.05, **p < 0.01, ***p < 0.001 vs. S. [file JCSM-11-1779-s003.tiff]

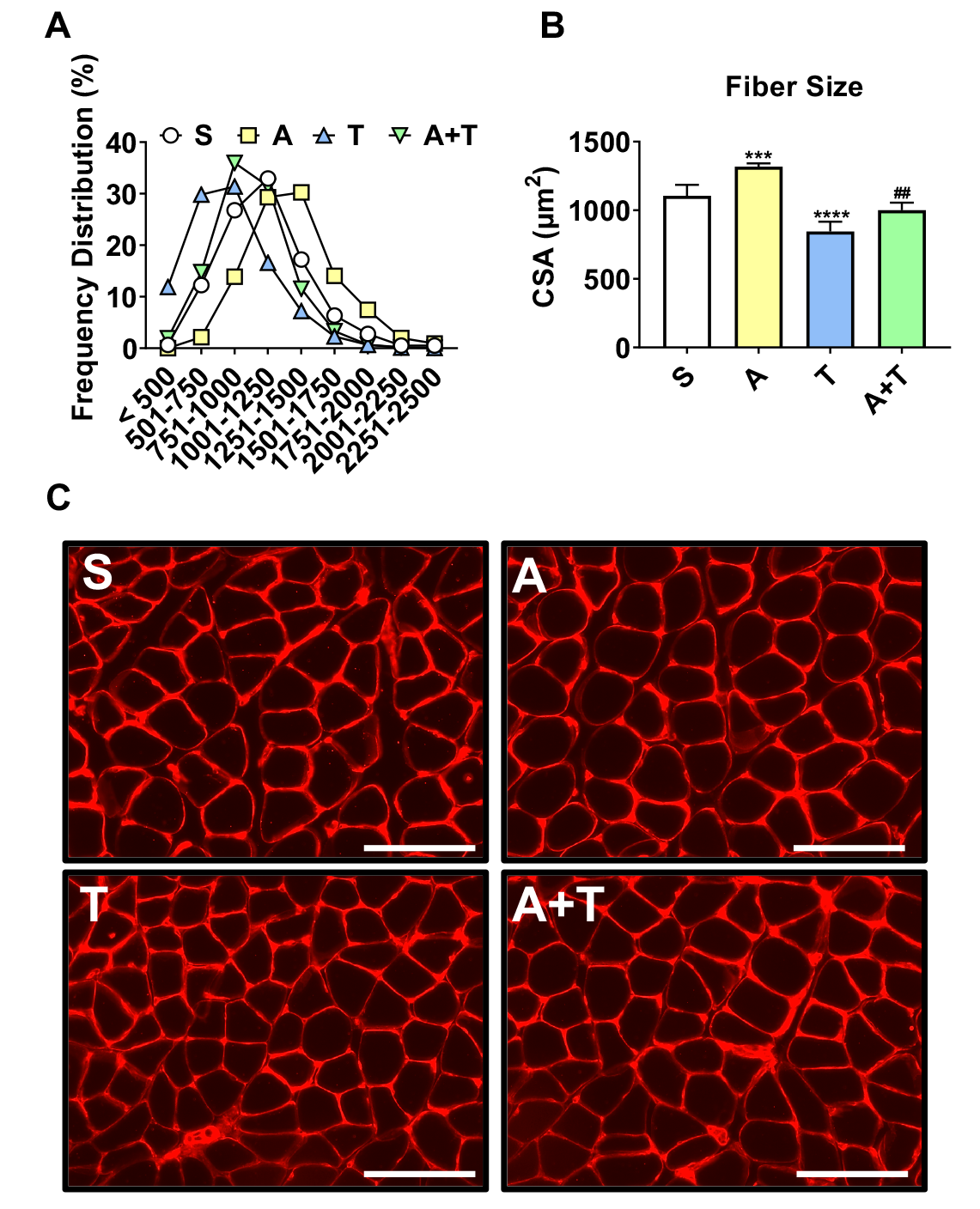

Supplement: Supplementary file 4 — Figure S4 ACVR2B/Fc preserves fiber size of soleus muscles in mHCT116 hosts. (A) Cross‐sectional area (CSA) frequency distribution, (B) mean CSA, and (C) representative images of laminin stained soleus muscles to assess CSA in NSG male mice (8‐week old) intrasplenically injected with HCT116 tumor cells (1.25 × 10^5^ cells/mouse in sterile PBS: T) or an equal volume of vehicle (Sham: S) and administered ACVR2B/Fc (A) (n = 4–6). Images were taken at 20× magnification. Scale bars: 100 μm. Data are expressed as mean ± SD. Significance of the differences: ***p < 0.001, ****p < 0.0001 vs. S; ##p < 0.01 vs. T. [file JCSM-11-1779-s004.tiff]

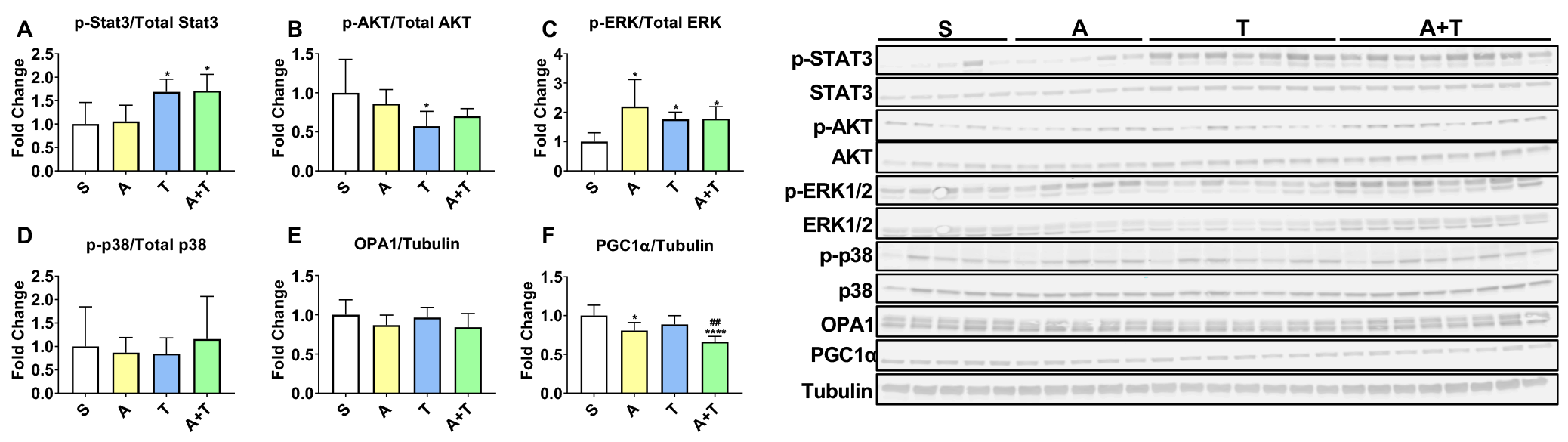

Supplement: Supplementary file 9 — Figure S9 ACVR2B/Fc does not improve markers of anabolism or catabolism in the heart of mHCT116 hosts. Representative western blotting and quantification (expressed as fold change versus sham) for (A) phospho‐Stat3, Stat3, (B) phospho‐AKT, AKT, (C) phospho‐ERK1/2, ERK1/2, (D) phospho‐p38, p38, (E) OPA1, (F) PGC1α and tubulin in heart tissue from NSG male mice (8‐week old) intrasplenically injected with HCT116 tumor cells (1.25 × 10^5^ cells/mouse in sterile PBS: T) or an equal volume of vehicle (Sham: S) and administered ACVR2B/Fc (A) (n = 5–8). Data are expressed as mean ± SD. Significance of the differences: *p < 0.05 vs. S. [file JCSM-11-1779-s009.tiff]

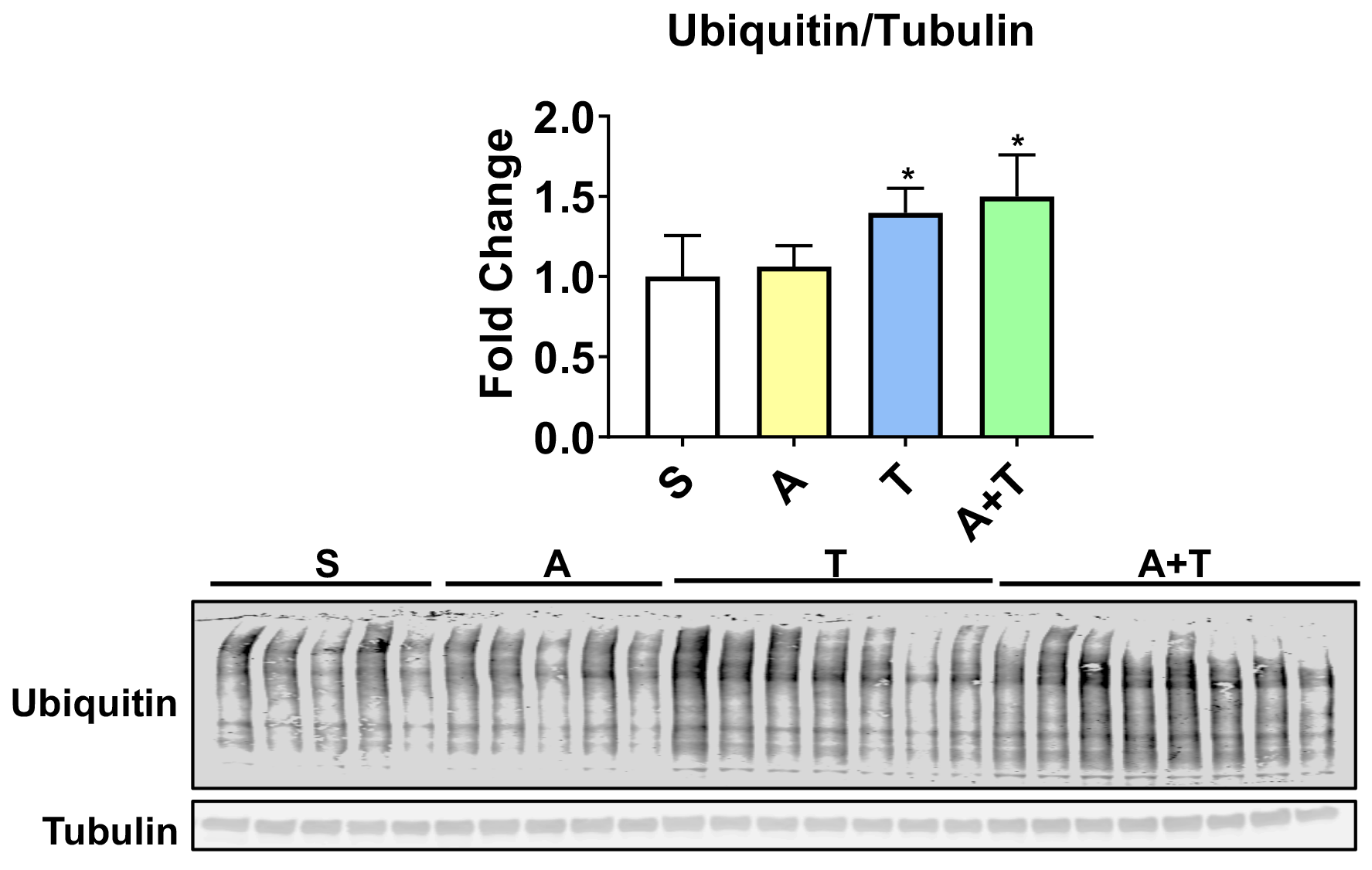

Supplement: Supplementary file 10 — Figure S10 Heart Ubiquitin does not improve with ACVR2B/Fc in mHCT116 hosts. Representative western blotting and quantification (expressed as fold change versus sham) for total ubiquitin and tubulin from heart tissue of 8‐week‐old NSG male mice (8‐week old) intrasplenically injected with HCT116 tumor cells (1.25 × 10^5^ cells/mouse in sterile PBS: T) or an equal volume of vehicle (Sham: S) and administered ACVR2B/Fc (A) (n = 5–8). Data are expressed as mean ± SD. Significance of the differences: *p < 0.05 vs. S. [file JCSM-11-1779-s010.tiff]
